# The Heck reaction of allylic alcohols catalysed by an N-heterocyclic carbene-Pd(ii) complex and toxicity of the ligand precursor for the marine benthic copepod *Amphiascoides atopus*†

**DOI:** 10.1039/d1ra03484g

**Published:** 2021-06-07

**Authors:** Jorge Cárdenas, Ruben Gaviño, Eréndira García-Ríos, Lucero Rios-Ruiz, Ana C. Puello-Cruz, Francisco Neptalí Morales-Serna, Samuel Gómez, Adolfo López-Torres, José Antonio Morales-Serna

**Affiliations:** Instituto de Química, Universidad Nacional Autónoma de México Circuito Exterior, Ciudad Universitaria Ciudad de México 04510 México; Centro de Investigación en Alimentación y Desarrollo A.C., Unidad Académica Mazatlán en Acuicultura y Manejo Ambiental Mazatlán Sinaloa 82112 México; Instituto de Ciencias del Mar y Limnología, Universidad Nacional Autónoma de México Mazatlán Sinaloa 82040 México neptali@ola.icmyl.unam.mx; Instituto de Química Aplicada, Universidad del Papaloapan Tuxtepec Oaxaca 68301 México joseantonio.moralesserna@gmail.com

## Abstract

The palladium-catalysed reaction of aryl halides and allylic alcohols is an attractive method for obtaining α,β-unsaturated aldehydes and ketones, which represent key intermediates in organic synthesis. In this context, a 1,2,3-triazol-5-ylidene (aNHC)-based palladium(ii) complex formed *in situ* has been found to be a selective catalyst for the syntheses of building blocks from the corresponding aryl halides and allylic alcohols, with yields ranging from 50% to 90%. The lack of toxic effects of the ligand precursor (1,2,3-triazolium salt) of the palladium(ii) complex for the harpacticoid copepod *Amphiascoides atopus* allowed us to contrast the efficiency of the catalytic system with the potential impact of the principal waste chemical in global aquatic ecosystems, which has not been previously addressed.

## Abstract 2

A 1,2,3-triazol-5-ylidene (aNHC)-based palladium(ii) complex formed *in situ* has been found to be an efficient catalyst in the formation of C–C bonds.

## Graphical abstract

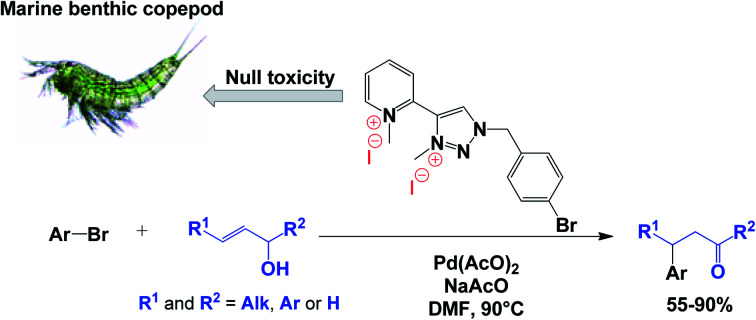

## Introduction

Easy access to 1,2,3-triazoles *via* a simple copper-catalysed azide–alkyne cycloaddition reaction (CuAAC) has led to the development of a “post-click chemistry” strategy to obtain 1,2,3-triazolium salts by selective alkylation at the N3 position of the triazole ring.^1^ 1,2,3-Triazolium salts represent an attractive group of chemical compounds given that they can be used as ionic liquids (ILs),^2^ as hosts in anion recognition, as components of molecular machines and supramolecular assemblies^3^ or as precursors of abnormal (mesoionic)^4^ N-heterocyclic carbenes (aNHCs) as a consequence of their high stability.^5^

**Scheme 1:**
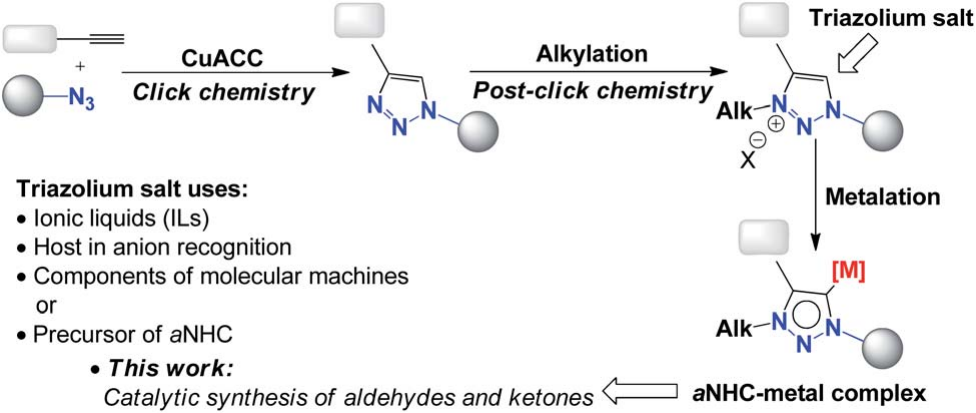
Synthesis and uses of 1,2,3-triazolium salt.

As shown in Scheme 1, deprotonation and metalation of 1,2,3-triazolium salts give a 1,2,3-triazol-5-ylidene complex (aNHC), which may be a precursor for an efficient catalyst for organic reactions thanks to its unique electronic features and donor properties.^6^ Recently, 1,2,3-triazolium iodide salts have been used as efficient precursors for 1,2,3-triazol-5-ylidene ligands^7^ (aNHCs) for palladium-catalysed Suzuki–Miyaaura^8^ and Heck–Mizoroki cross-coupling reactions.^9^ In those cases, the aNHC and palladium(ii) complex are formed *in situ* from triazolium salts under mild reaction conditions. With these relevant protocols, we focused on the use of a similar system in the synthesis of α–β unsaturated aldehydes and ketones *via* the Heck coupling of aryl halides and allylic alcohols, with the idea of developing an efficient and selective protocol using 1,2,3-triazol-5-ylidene (aNHC)-based palladium(ii) complexes.

The high thermal stability and low vapour pressure make 1,2,3-triazolium salts attractive for industrial eco-friendly processes. It is assumed that their structural characteristics do not cause air pollution or damage to occupational health. However, their properties, such as resistance to photodegradation, water solubility and stability, suggest that they may be a threat to aquatic ecosystems due to bioaccumulation.^10^ For that reason, we considered it necessary to compare the efficiency of the catalytic system (aNHC-based palladium(ii) complexes) with the impact of the 1,2,3-triazolium salt precursor in aquatic ecosystems.^11^ Thus, we evaluated its acute toxicity in the harpacticoid copepod *Amphiascoides atopus*, considering that it will be among the principal waste chemicals eliminated after the catalytic process.

Sediments in aquatic ecosystems are rich in small crustaceans, including benthic invertebrates, which are effective indicators of impacts at higher levels of biological organization given their importance to overall ecosystem structure and function.^12^ In particular, marine copepods belonging to the order Harpacticoida, one of the most abundant benthic invertebrates and an important food source for macroinvertebrates and fish, are suitable for use in tests that rapidly assess the acute, sublethal, or chronic effects of contaminants.^13^ To our knowledge, there are no previous studies testing the toxicity of 1,2,3-triazolium salts in copepods or in other crustaceans.

## Results and discussion

### Catalytic activity

**Scheme 2:**
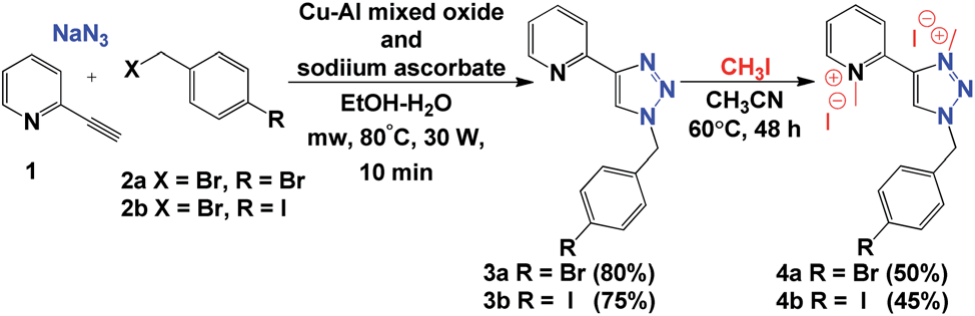
Synthesis of 1,3,4-trisubstituted-1,2,3-triazolium iodide salts 4a and 4b.

Initially, we carried out the synthesis of 1,2,3-triazolium iodide salts 4a and 4b with a simple copper-catalysed azide–alkyne cycloaddition (CuAAC) of alkyne 1, bromide derivatives 2a and 2b, and NaN_3_ under heterogeneous catalytic conditions. Then, direct methylation of both nitrogen atoms of the triazole and pyridine ring allowed us to obtain 1,3,4-trisubstituted-1,2,3-triazolium iodide salts 4a and 4b (Scheme 2).

Then, we were interested in evaluating the catalytic properties of aNHC-based palladium(ii) complexes formed *in situ* from 4a and 4b in the Heck reaction between bromo pyridine 5a and allylic alcohol 6a to give ketone 7a. After various solvent systems were examined, we concluded that DMF was the best solvent (Table 1, entries 1–6). A higher reaction yield was obtained in the presence of Pd(OAc)_2_ and NaOAc (Table 1, entry 1). Furthermore, the yield was lowered when the reaction was carried out in the presence of PdCl_2_ (Table 1, entries 2 and 4) or when *t*-BuOK or K_2_CO_3_ was used as the base (Table 1, entries 7 and 8). When the reaction was performed in THF under reflux, only the starting material was observed in the ^1^H NMR spectrum of the reaction mixture (Table 1, entry 5). Product 7a was obtained in 55% and 50% yields at 50 °C (Table 1, entry 9), and it was not observed at room temperature (Table 1, entry 10) in the presence of 4a or 4b. When the reaction was carried out with 5 mol% 4a or 4b and 5 mol% Pd(OAc)_2_, the yield did not increase considerably (Table 1, entry 11). Notably, a complex reaction mixture was obtained in the absence of triazolium salts 4a or 4b.

**Table 1:** Coupling of aryl halide 5a with allylic alcohol 6b in the presence of 4a or 4b^a^

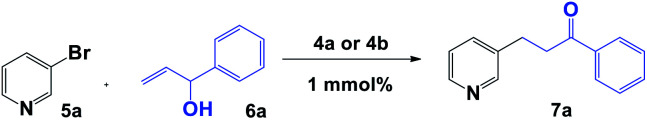					
Entry	Solvent	Pd(ii) source	Base	*T* (°C)	Yield^b^ (%)
1	DMF	Pd(OAc)_2_	NaOAc	90	80^c^ and 78^d^
2	DMF	PdCl_2_	NaOAc	90	65^c^ and 60^d^
3	Toluene	Pd(OAc)_2_	NaOAc	90	67^c^ and 60^d^
4	Toluene	PdCl_2_	NaOAc	90	48^c^ and 45^d^
5	THF	Pd(OAc)_2_	NaOAc	Reflux	0^c^ and 0^d^
6	H_2_O	Pd(OAc)_2_	NaOAc	90	15^c^ and 15^d^
7	DMF	Pd(OAc)_2_	*t*-BuOK	90	70^c^ and 60^d^
8	DMF	Pd(OAc)_2_	K_2_CO_3_	90	72^c^ and 62^d^
9	DMF	Pd(OAc)_2_	NaOAc	50	55^c^ and 50^d^
10	DMF	Pd(OAc)_2_	NaOAc	rt	0^c^ and 0^d^
11^e^	DMF	Pd(OAc)_2_	NaOAc	90	82^c^ and 80^d^

a Reaction conditions: aryl halide 5a (1.0 mmol), allylic alcohol 6a (1.0 mmol), base (2.0 mmol), Pd(ii) source (1 mol%), 1,2,3-triazolium salt 4 (1 mol%) and solvent (5 mL).

b Yields of isolated product after chromatographic purification.

c Yield obtained with 4a as aNHC precursor.

d Yield obtained with 4b as aNHC precursor.

e 5% of Pd(OAc)_2_ and 5% of 1,2,3-triazolium salt 4 were used.

Using the best reaction conditions, we next examined the application of a 1,2,3-triazolium iodide and palladium salt system from the 4a aNHC precursor for the cross-couplings of a variety of aryl bromides with primary or secondary allylic alcohols (Table 2). The results indicate that the aNHC-based palladium(ii) complex formed *in situ* constituted an efficient and selective catalyst system for the synthesis of saturated aldehydes 7b–7e and ketones 7f–7r in good yields. In the case of tertiary allylic alcohols, the only final products were α–β unsaturated alcohols (7s–7v, Table 2). Unfortunately, the reaction does not evolve successfully when it was carried with aryl chlorides. One of the principal drawbacks of the reaction studied here is the formation of mixtures with both saturated and unsaturated carbonyl compounds as principal products,^14,15^ which was not observed under our reaction conditions. Saturated carbonyl compounds were obtained as the only product, and unsaturated products (α–β unsaturated carbonyl compounds) were not observed when crude products were analysed by NMR.

**Table 2:** Scope of N-heterocyclic carbene and Pd(ii) complex-catalysed allylic alcohol Heck reaction^a^^,^^b^

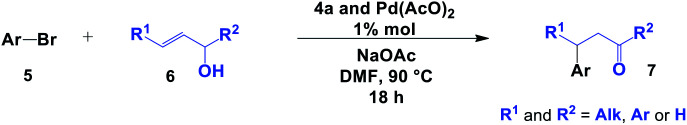
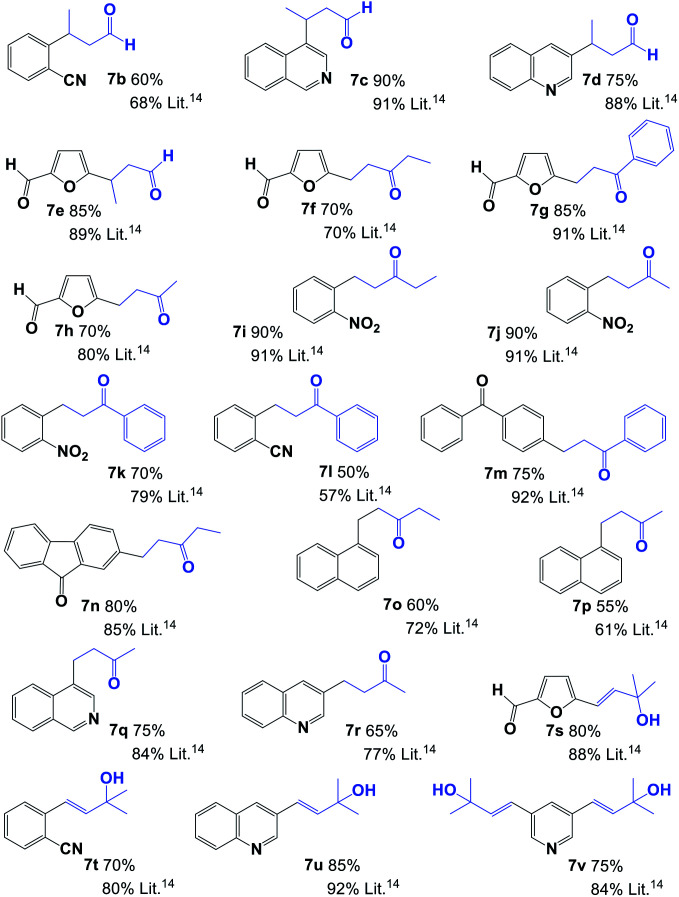

a Reaction conditions: aryl halide 5 (1.0 mmol), allylic alcohol 6 (1.0 mmol), NaOAc (2.0 mmol), Pd(OAc)_2_ (1 mol%), and 1,2,3-triazolium salt 4a (1 mol%) and DMF (5 mL).

b Yields of isolated product after chromatographic purification.

**Scheme 3:**
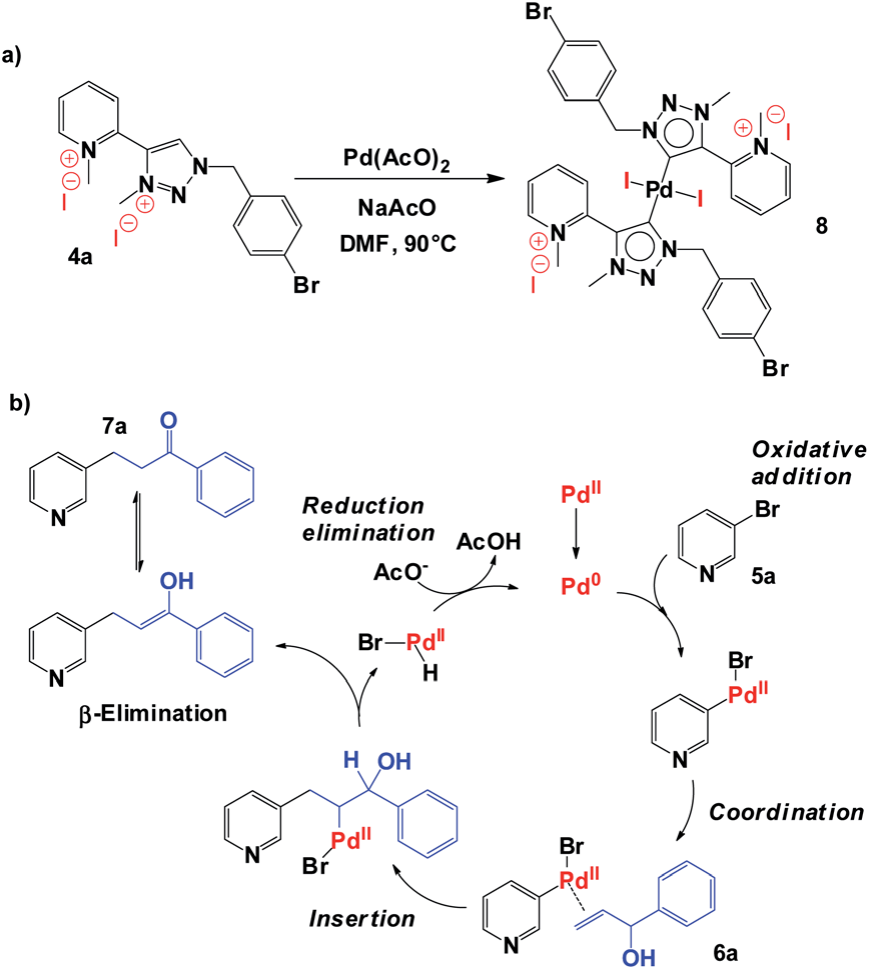
(a) Proposed structure of 1,2,3-triazol-5-ylidene (aNHC)-based palladium(ii) complex 8. (b) Proposed mechanism for synthesis of α–β unsaturated aldehydes and ketones *via* the Heck coupling of aryl halide 5a and allylic alcohol 6a.

Regarding the reaction mechanism, we propose the direct metalation of 1,2,3-triazolium salt 4a with Pd(AcO)_2_ in the presence of NaOAc to obtain *in situ* 1,2,3-triazol-5-ylidene (aNHC)-based palladium(ii) complexes 8 (Scheme 3a), which should be the principal precursor to the catalytic species in the reaction. The catalytic process starts with the oxidative addition of bromo pyridine 5a, followed by coordination and regioselective alkene insertion (allylic alcohol 6a). Finally, Pd–H β-elimination involving carbinol hydrogen furnished desired carbonyl compound 7a.^16^

### Toxicity of 1,2,3-triazolium salt 4a

**Fig. 1:**
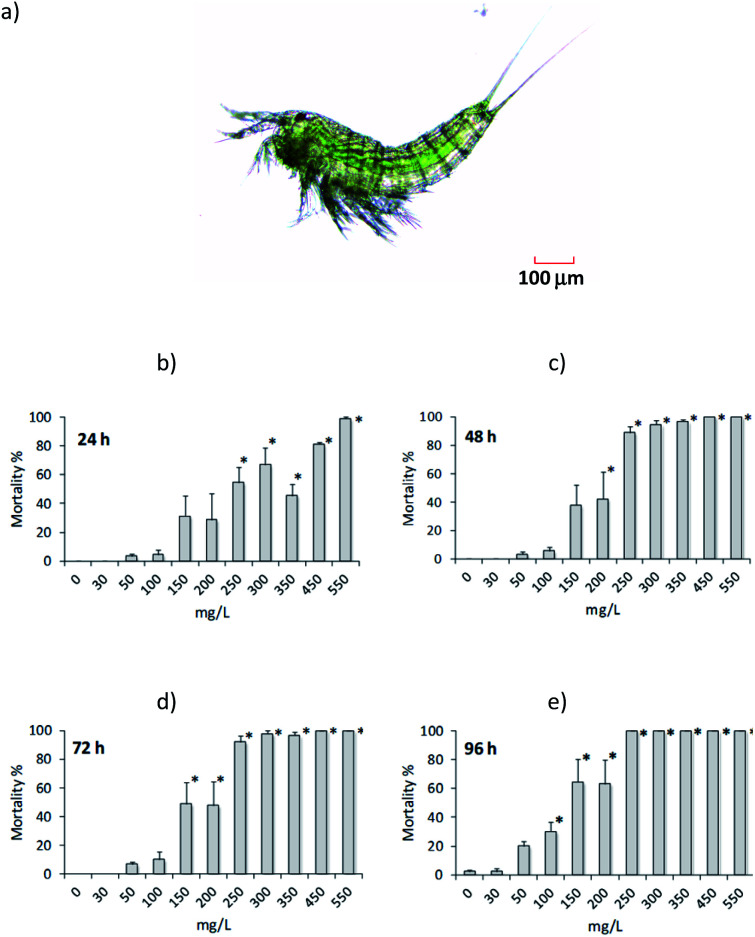
Toxicity of 1,2,3-triazolium salt 4a against (a) the harpacticoid copepod *Amphiascoides atopus* at (b) 24 h, (c) 48 h, (d) 72 h and (e) 96 h. Asterisk (*) indicates significant difference from controls.

Then, we used the harpacticoid copepod *Amphiascoides atopus* as a model to evaluate the toxicity (LC50 at 24, 48, 72 and 96 h) of 1,2,3-triazolium salt 4a. The results obtained were based on the mortality of adult copepods, which varied significantly among triazolium salt concentrations at every exposure time (Fig. 1, ANOVA: *F* = 18.156 for 24 h, *F* = 39.54 for 48 h, *F* = 45.562 for 72 h, and *F* = 34.951 for 96 h; all *P* < 0.0001). There was practically no mortality in unexposed (control) organisms and those exposed to 30 mg L^−1^4a. Some mortality was observed at higher concentrations (Fig. 1); however, compared to the control, significant differences occurred above 250 mg L^−1^ at 24 h, 200 mg L^−1^ at 48 h, 150 mg L^−1^ at 72 h, and 100 mg L^−1^ at 96 h (all *P* < 0.05). The estimated LC50 values were 250.4 mg L^−1^ (CI 215.4 to 290.5 mg L^−1^) at 24 h, 173.9 mg L^−1^ (CI 150.4 to 196.4 mg L^−1^) at 48 h, 155 mg L^−1^ (CI 135.6 to 173.9 mg L^−1^) at 72 h, and 111.5 mg L^−1^ (CI 93.4 to 129.8 mg L^−1^) at 96 h. From these results and following the hazard classification of the US EPA adopted by Tsarpali and Dailianis^17^ for crustaceans, the triazolium salt tested in the present study may be considered practically nontoxic since the estimated LC50 values were >100 mg L^−1^ at every exposure time. Similarly, Rodriguez Castillo *et al.*^18^ reported no toxicity of [BMTriaz][NTf_2_] and [CF_3_CF_2_BTriaz][NTf_2_] triazolium salts against zebrafish *Danio rerio*.

The lack of toxic effect of triazolium salt 4a tested herein could be attributed to its hydrophilic properties, which limit its biocompatibility and adsorption onto or intercalation into the cellular membrane.^19^ Our results are based only on the mortality of adult copepods. This may be a shortcoming considering that early developmental stages of copepods as well as sublethal endpoints are more sensitive indicators of the toxicity of contaminants.^20^ Additionally, further studies are required to test the effect of the triazolium salt used in the present work on other aquatic organisms, including marine and freshwater species. However, it is noteworthy that there are no previous studies testing the toxicity of triazolium salts in marine eukaryotes or copepods because we consider that the LC50 values from toxicity bioassays described in the present work are fundamental for future more ecologically relevant studies.^21^

## Conclusions

In summary, a 1,2,3-triazol-5-ylidene (aNHC)-based palladium(ii) complex, formed *in situ* from a 1,2,3-triazolium salt, is an efficient catalytic system for the formation of C–C bonds (Heck coupling) in the selective synthesis of α–β unsaturated aldehydes and ketones from the corresponding aryl halides and allylic alcohols. The efficiency of this catalytic system is reinforced by the null toxicity of the catalytic precursor (1,2,3-triazolium salt) in the marine benthic copepod *Amphiascoides atopus*. This result, in addition to the high thermal stability and low vapour pressure of the catalytic precursor, allows a glimpse of the low impact in air and in aquatic ecosystems resulting from one of the main waste chemicals produced in the catalytic process studied here.

## Experimental section

### General methods

All reagents were purchased from Aldrich Chemical Co and used without further purification unless stated otherwise. Yields refer to the chromatographically and spectroscopically (^1^H and ^13^C) homogeneous materials. The organic reactions were monitored by TLC carried out on 0.25 mm E. Merck silica gel plates. The developed TLC plates were visualised under a short-wave UV lamp or by heating after they were dipped in Ce(SO_4_)_2_. Flash column chromatography (FCC) was performed using silica gel (230–400) and employed a solvent polarity correlated with the TLC mobility. NMR experiments were conducted on a Varian 300 and Bruker 500 MHz instruments in CDCl_3_ (99.9% D) and CD_3_OD (99.8% D) as solvents; the chemical shifts (*δ*) were referenced to CHCl_3_ (7.26 ppm ^1^H, 77.00 ppm ^13^C), CD_3_OD (4.87 ppm ^1^H, 49.00 ppm ^13^C), or TMS (0.00 ppm). The chemical shifts are reported in parts per million (ppm).

### Toxicity test

Copepods (*A. atopus*) were obtained from a stock maintained in laboratory culture in a 15 L flask with filtered natural seawater (35‰) under natural conditions of photoperiod (typically, 12 h light/12 h dark) and temperature (28 ± 0.5 °C), and fed with microalgae diet.^22^

To choose the test concentrations a range-finding test was performed. The definitive tests were performed in six-well culture plates. Ten copepods (ovigerous females) were randomly transferred to each well containing 5 mL of filtered seawater (35‰) at 28 ± 0.5 °C. Copepods were not fed and exposed to increasing concentrations of 1,3,4-trisubstituted-1,2,3-triazolium iodide salts 4a: 0, 30, 50, 100, 150, 200, 250, 300, 350, 450 and 550 mg L^−1^ for 96 h. Experiments were set up with six replicates. Mortality of copepods was checked under a stereomicroscope every 24 h. Copepods were considered dead if they did not show movement of appendages and did not show any reaction when transferred to wells with seawater without 1,3,4-trisubstituted-1,2,3-triazolium iodide salts 4a in a period of up to 20 s of observation.

Significant differences in mortality among different concentrations of 1,3,4-trisubstituted-1,2,3-triazolium iodide salts 4a were determined by one-way ANOVA with the Holm–Sidak test posteriori, performed in RStudio software. Significant differences were considered when *P* < 0.05. The LC50 value and its respective 95% confidence interval were determined by probit analysis method (Finney, 1971) using PASW Statistics 18 software (SPSS Inc. Chicago, IL, USA).

### General procedure

#### Synthesis of 1,2,3-triazoles 3a and 3b^23^

To a solution of alkyne 1 (1 mmol), benzyl halide 2a or 2b (1.2 mmol), NaN_3_ (1.2 mmol) in a mixture of THF–H_2_O (20 mL, 1 : 1 v/v), were added sodium ascorbate (0.5 mmol%) and CuSO_4_ (0.5 mmol%). The mixture was stirred at 50 °C for 48 h. After the reaction time, the mixture was cooled to room temperature and THF was eliminated under vacuum. The resulting precipitate of the Cu complex was decomposed by addition of small portions of aqueous ammonia. Then, the mixture was diluted with ethyl acetate (25 mL), washed with NaHCO_3_ (7% aqueous, 2 × 10 mL) and brine (2 × 10 mL), dried over Na_2_SO_4_ and concentrated under vacuum. The crude product was purified through a silica gel column chromatography with a gradient AcOEt–hexane.

#### Synthesis of 1,2,3-triazolium salts 4a and 4b

A mixture of 1,2,3-triazole 3a or 3b (1.0 mmol) and CH_3_CN (10 mL) was treated with CH_3_I (20.0 mmol) at 60 °C for 48 h with vigorous stirring. Then, the white precipitate obtained was filtered and washed with acetonitrile and dried under vacuum to obtain the corresponding 1,2,3-triazolium iodide salts 4a or 4b.

### General procedure for the catalytic reactions

In all Heck reactions, a DMF solution (5 mL) of 1 mmol of aryl halide 5, 1.0 mmol of allylic alcohol 6, 2.0 mmol of NaOAc, 1% mmol of Pd(OAc)_2_ and 1% mmol of 1,2,3-triazolium salt 4 were heated for 6 h in a 90 °C silicon oil bath equipped with a condenser system. After the reaction time, the mixture was cooled to room temperature and diluted with ethyl acetate (15 mL), washed with brine (3 × 10 mL), dried over Na_2_SO_4_ and concentrated under vacuum. The crude product was purified through a silica gel column chromatography with a gradient AcOEt–hexane.

### Characterization data

#### 2-(1-(4-Bromobenzyl)-1*H*-1,2,3-triazol-4-yl)pyridine 3a (80%)

^1^H (CDCl_3_, 500 MHz): *δ* 8.52 (ddd, *J* = 4.5, 1.5, 1.0 Hz, 1H), 8.16 (ddd, *J* = 8.0, 1.5, 1.0 Hz, 1H), 8.03 (brs, 1H), 7.75 (ddd, *J* = 8.0, 7.5, 1.5 Hz, 1H), 7.35 (m, 4H), 7.19 (ddd, *J* = 7.5, 4.5, 1.5 Hz, 1H), 5,57 (brs, 2H). NMR ^13^C (CDCl_3_, 125 MHz): *δ* 150.4, 149.4, 148.5, 137.0, 134.5, 129.3, 128.9, 128.4, 122.97, 122.04, 120.03, 54.5. Anal. calcd for C_14_H_11_BrN_4_: C, 53.35; H, 3.52; N, 17.78. Found: C, 53.30; H, 3.48; N, 17.73.

#### 2-(1-(4-Iodobenzyl)-1*H*-1,2,3-triazol-4-yl)pyridine 3a (75%)

^1^H (CDCl_3_, 500 MHz): *δ* 8.54 (ddd, *J* = 4.5, 1.5, 1.0 Hz, 1H), 8.17 (ddd, *J* = 8.0, 1.5, 1.0 Hz, 1H), 8.04 (brs, 1H), 7.76 (ddd, *J* = 8.0, 7.5, 1.5 Hz, 1H), 7.71 (AA′BB′, m, 2H), 7.21 (ddd, *J* = 7.5, 4.5, 1.5 Hz, 1H), 7.07 (AA′BB′, m, 2H), 5.52 (brs, 2H). NMR ^13^C (CDCl_3_, 125 MHz): *δ* 150.2, 149.5, 149.1, 138.4, 137.0, 134.2, 130.1, 123.0, 122.0, 120.3, 94.81, 53.9. Anal. calcd for C_14_H_11_IN_4_: C, 46.43; H, 3.06; N, 15.47. Found: C, 46.37; H, 3.01; N, 15.42.

#### 2-(1-(4-Bromobenzyl)-3-methyl-1*H*-1,2,3-triazol-3-ium-4-yl)-1-methylpyridin-1-ium iodide 4a (50%)

^1^H (CD_3_OD, 500 MHz): *δ* 8.98 (brs, 1H), 8.97 (m, 1H), 8.59 (m, 1H), 8.49 (dd, *J* = 8.0, 1.5 Hz, 1H), 8.02 (ddd, *J* = 7.5, 6.0, 1.5 Hz, 1H), 7.57 (AA′BB′, m, 2H), 7.39 (AA′BB′, m, 2H), 5,57 (brs, 2H), 4.56 (brs, 3H), 3.31 (brs, 1H). NMR ^13^C (CD_3_OD, 125 MHz): *δ* 148.2, 146.4, 140.2, 135.2, 133.2, 131.3, 129.9, 129.8, 129.6, 127.6, 123.8, 54.6, 49.2, 49.1. Anal. calcd for C_16_H_17_BrI_2_N_4_: C, 32.08; H, 2.86; Br, N, 9.35. Found: C, 32.01; H, 2.82; N, 9.30.

#### 2-(1-(4-Iodobenzyl)-3-methyl-1*H*-1,2,3-triazol-3-ium-4-yl)-1-methylpyridin-1-ium iodide 4b (45%)

^1^H (CD_3_OD, 500 MHz): *δ* 8.98 (brs, 1H), 8.97 (m, 1H), 8.59 (m, 1H), 8.49 (dd, *J* = 8.0, 1.5 Hz, 1H), 8.02 (ddd, *J* = 7.5, 6.0, 1.5 Hz, 1H), 7.57 (AA′BB′, m, 2H), 7.39 (AA′BB′, m, 2H), 5,57 (brs, 2H), 4.58 (s, 3H), 4.56 (s, 3H). NMR ^13^C (CD_3_OD_3_, 125 MHz): *δ* 148.2, 146.5, 140.3, 135.2, 133.1, 131.4, 131.1, 129.9, 129.6, 127.6, 123.8, 54.7, 49.3, 49.2. Anal. calcd for C_16_H_17_I_3_N_4_: C, 29.75; H, 2.65; Br, N, 8.67. Found: C, 29.71; H, 2.59; N, 8.62.

#### 1-Phenyl-3-(pyridin-3-yl)propan-1-one 7a (80% yield)

NMR ^1^H (CDCl_3_, 300 MHz): *δ* 8.45 (brs, 1H), 8.35 (d, *J* = 3.7 Hz, 1H), 7.86 (d, *J* = 7.0 Hz, 2H), 7.52 (m, 4H), 7.14 (dd, *J* = 7.5, 6.1 Hz, 1H), 3.19 (t, *J* = 7.2 Hz, 2H), 2.98 (t, *J* = 7.2 Hz, 2H). NMR ^13^C (CDCl_3_, 75 MHz): *δ* 198.1, 149.3, 146.9, 136.5, 136.2, 136.1, 133, 128.5, 127.7, 123.2, 39.3, 26.7. Anal. calcd for C_14_H_13_NO: C, 79.59; H, 6.20; N, 6.63. Found: C, 79.55; H, 6.17; N, 6.61.

#### 2-(1-Methyl-3-oxopropyl)benzonitrile 7b (60% yield)

NMR ^1^H (CDCl_3_, 300 MHz): *δ* 9.84 (t, *J* = 1.4 Hz, 1H), 7.62 (m, 2H), 7.18 (m, 2H), 3.68 (sex, *J* = 7.2 Hz, 1H), 2.75 (dd, *J* = 16, 7.2 Hz, 1H), 2.66 (dd, *J* = 16, 7.2 Hz, 1H), 1.34 (d, *J* = 7.2 Hz, 3H). NMR ^13^C (CDCl_3_, 75 MHz): *δ* 200.3, 148.9, 133.3, 133, 127, 126.4, 117.8, 112, 50.5, 32.4, 21.4. Anal. calcd for C_11_H_11_NO: C, 76.28; H, 6.40; N, 8.09. Found: C, 76.25; H, 6.34; N, 8.01.

#### 3-(Isoquinolin-4-il)butanal 7c (90% yield)

NMR ^1^H (CDCl_3,_ 300 MHz): *δ* 9.85 (t, *J* = 1.4 Hz, 1H), 9.16 (brs, 1H), 8.47 (brs, 1H), 8.09 (dd, *J* = 8.2, 1.1 Hz, 1H), 8.01 (dd, *J* = 8.0, 1.0 Hz, 1H), 7.78 (ddd, *J* = 8.6, 6.8, 1.4 Hz, 1H), 7.64 (ddd, *J* = 8.2, 6.8, 1.0 Hz, 1H), 4.15 (sex, *J* = 6.8 Hz, 1H), 2.99 (ddd, *J* = 16.1, 5.6, 1.2 Hz, 1H), 2.89 (ddd, *J* = 16.8, 5.7, 1.4 Hz, 1H), 1.48 (d, *J* = 6.8 Hz, 1H). NMR ^13^C (CDCl_3_, 75 MHz): *δ* 200.9, 151.7, 140.1, 134.5, 133.7, 130.6, 128.6, 128.45, 126.9, 121.9, 50.8, 26.9, 21.4. Anal. calcd for C_13_H_13_NO: C, 78.36; H, 6.58; N, 7.03. Found: C, 78.27; H, 6.47; N, 6.96.

#### 3-(Quinolin-3-yl)butanal 7d (75% yield)

NMR ^1^H (CDCl_3_, 300 MHz): *δ* 9.73 (t, *J* = 1.4, 1H), 8.81 (d, *J* = 2.3, 1H), 8.08 (d, *J* = 7.0, 1H), 7.97 (d, *J* = 2.3, 1H), 7.78 (d, *J* = 7.0, 1H), 7.64 (d, *J* = 7.0, 1H), 7.56 (d, *J* = 7.1, 1H), 3.57 (sex, *J* = 7.2 Hz, 1H), 2.88 (ddd, *J* = 17.2, 6.8, 1.4, 1H, Hz), 2.76 (ddd, *J* = 17.2, 7.4, 1.4 Hz, 1H), 1.40 (d, *J* = 7.0 Hz, 3H). NMR ^13^C (CDCl_3_, 75 MHz, DEPT): *δ* 200.6, 150.5, 146.8, 137.9, 132.8, 128.9, 127.9, 127.5, 126.7, 51.2, 31.5, 21.8. Anal. calcd for C_13_H_13_NO: C, 78.36; H, 6.58; N, 7.03. Found: C, 78.30; H, 6.51; N, 6.95.

#### 5-(4-Oxobutan-2-yl)furan-2-carbaldehyde 7e (85% yield)

NMR ^1^H (CDCl_3,_ 300 MHz): *δ* 9.79 (t, *J* = 1.6 Hz, 1H), 9.54 (brs, H1), 7.17 (d, *J* = 3.6 Hz, 1H), 6.29 (d, *J* = 3.6 Hz, 1H), 3.54 (m, 1H), 2.95 (ddd, *J* = 17.6, 6.4, 1.6 Hz, 1H), 2.72 (ddd, *J* = 17.6, 7.2, 1.6 Hz, 1H), 1.37 (d, *J* = 7.2 Hz, 3H). NMR ^13^C (CDCl_3_, 75 MHz): *δ* 199.7, 177.2, 167.3, 151.9, 123.2, 107.9, 48.5, 27.8, 18.5. Anal. calcd for C_9_H_10_O: C, 65.05; H, 6.07. Found: C, 64.96; H, 5.93.

#### 5-(3-Pentanona)furan-2-carbaldehyde 7f (70% yield)

NMR ^1^H (CDCl_3_, 300 MHz): *δ* 9.65 (s, 1H), 7.33 (d, *J* = 3.6 Hz, 1H, H-3), 6.43 (d, *J* = 3.6 Hz, 1H), 3.16 (t, *J* = 6.8 Hz, 2H), 3.02 (t, *J* = 6.8 Hz, 2H), 2.62 (q, *J* = 7.2 Hz, 2H), 1.21 (t, *J* = 7.4, 3H). NMR ^13^C (CDCl_3_, 75 MHz): *δ* 208.9, 176.7, 161.9, 151.7, 123.6, 109.1, 39.2, 35.7, 22.2, 7.5. Anal. calcd for C_10_H_12_O_3_: C, 66.65; H, 6.71. Found: C, 66.61; H, 6.67.

#### 5-(3-Oxo-3-phenylpropyl)furan-2-carbaldehyde 7g (85% yield)

NMR ^1^H (CDCl_3_, 300 MHz): *δ* 9.41 (brs, 1H), 7.87 (d, *J* = 7.5 Hz, 2H), 7.41 (m, 3H), 7.11 (d, *J* = 3.6 Hz, 1H), 6.25 (d, *J* = 3.5 Hz, 1H), 3.32 (t, *J* = 7.2 Hz, 2H), 3.08 (t, *J* = 7.2 Hz, 2H). NMR ^13^C (CDCl_3_, 75 MHz): *δ* 197.4, 176.7, 162.1, 151.6, 135.9, 132.9, 128.4, 127.7, 123.7, 109.1, 35.7, 22.4. Anal. calcd for C_14_H_12_O_3_: C, 73.67; H, 5.30. Found: C, 73.62; H, 5.27.

#### 5-(2-Butanone)furan-2-carbaldehyde 7h (85% yield)

NMR ^1^H (CDCl_3,_ 200 MHz): *δ* 9.44 (brs, 1H), 7.16 (d, *J* = 3.6 Hz, 1H), 6.45 (d, *J* = 3.6 Hz, 1H), 2.94 (m, 2H), 2.82 (m, 2H), 2.11 (s, 3H). NMR ^13^C (CDCl_3_, 75 MHz): *δ* 206.1, 176.6, 161.8, 151.7, 123.5, 109.1, 40.5, 29.6, 22.1. Anal. calcd for C_9_H_10_O: C, 65.05; H, 6.07. Found: C, 64.95; H, 5.94.

#### 5-(2-Nitrobenzene)pentan-3-one 7i (90% yield)

(CDCl_3_, 300 MHz): *δ* 7.87 (d, *J* = 8.0 Hz, 1H), 7.55 (dd, *J* = 8.1 Hz, 7.3 Hz, 1H), 7.42 (m, 2H), 3.10 (t, *J* = 7.4 Hz, 2H), 2.78 (t, *J* = 7.6 Hz, 2H), 2.39 (q, *J* = 7.4 Hz, 2H), 1.01 (t, *J* = 7.4 Hz, 3H). NMR ^13^C (CDCl_3_, 75 MHz): *δ* 209.7, 149.1, 136.3, 133.1, 132.3, 127.3, 124.7, 42.6, 35.8, 27.1, 7.6. Anal. calcd for C_11_H_13_NO_3_: C, 63.76; H, 6.32; N, 6.76. Found: C, 63.70; H, 6.26; N, 6.71.

#### 4-(2-Nitrobenzene)butan-2-one 7j (90% yield)

NMR ^1^H (CDCl_3_, 300 MHz): *δ* 7.88 (d, *J* = 7.2 Hz, 1H), 7.51 (dd, *J* = 8.0 Hz, 7.4 Hz, 1H), 7.39 (m, 2H), 3.11 (t, *J* = 7.2 Hz, 2H), 2.85 (t, *J* = 7.2 Hz, 2H), 2.14 (s, 3H). NMR ^13^C (CDCl_3_, 75 MHz): *δ* 206.8, 148.9, 134.3, 133.0, 132.1, 127.2, 124.6, 43.8, 29.6, 26.9. Anal. calcd for C_10_H_11_NO_3_: C, 62.17; H, 5.74; N, 7.25. Found: C, 62.12; H, 5.70; N, 7.19.

#### 3-(2-Nitrobenzene)-1-phenylpropan-1-one 7k (70% yield)

NMR ^1^H (CDCl_3_, 300 MHz): *δ* 7.95 (m, 3H), 7.46 (m, 6H), 3.37 (m, 4H). NMR ^13^C (CDCl_3_, 75 MHz): *δ* 198.4, 149.2, 136.5, 136.4, 133.1, 132.5, 128.5, 127.9, 127.4, 124.8, 39.3, 27.6. Anal. calcd for C_15_H_13_NO_3_: C, 70.58; H, 5.13; N, 5.49. Found: C, 70.51; H, 5.05; N, 5.41.

#### 2-(3-Oxo-3-phenylpropyl)benzonitrile 7l (50% yield)

NMR ^1^H (CDCl_3_, 300 MHz): *δ* 7.95 (d, *J* = 7.8 Hz, 1H), 7.48 (m, 7H), 7.26 (t, *J* = 7.9 Hz, 1H), 3.32 (m, 4H). NMR ^13^C (CDCl_3_, 75 MHz): *δ* 197.9, 145.1, 136.3, 133.1, 132.8, 132.7, 129.9, 128.5, 127.9, 126.7, 117.9, 112.2, 38.9, 28.6. Anal. calcd for C_16_H_13_NO: C, 81.68; H, 5.57; N, 5.95. Found: C, 81.60; H, 5.51; N, 5.89.

#### 1-Phenyl-3-[4-(phenylcarbonyl)phenyl]propan-1-one 7m (75% yield)

NMR ^1^H (CDCl_3_, 300 MHz): *δ* 7.98 (d, *J* = 7.0 Hz, 2H), 7.79 (m, 4H), 7.45 (m, 8H), 3.35 (t, *J* = 7.2 Hz, 2H), 3.17 (t, *J* = 7.2 Hz, 2H). NMR ^13^C (CDCl_3_, 75 MHz): *δ* 198.7, 196.3, 146.4, 137.6, 136.5, 135.4, 133.1, 132.3, 130.4, 129.8, 128.5, 128.3, 128.1, 127.9, 39.7, 29.8. Anal. calcd for C_22_H_18_O_2_: C, 84.05; H, 5.77. Found: C, 83.98; H, 5.71.

#### 2-(3-Oxopentyl)-9*H*-fluorene-9-one 7n (80% yield)

NMR ^1^H (CDCl_3,_ 300 MHz): *δ* 7.62 (d, *J* = 7.9 Hz, 1H), 7.35 (m, 6H), 2.92 (t, *J* = 7.4 Hz, 2H), 2.76 (t, *J* = 7.4 Hz, 2H), 2.43 (q, *J* = 7.2 Hz, 2H), 1.05 (t, *J* = 7.4 Hz, 3H). NMR ^13^C (CDCl_3_, 75 MHz): *δ* 210.5, 194.5.8, 144.6, 142.8, 138.1, 136.9, 135.3, 134.5, 133.8, 124.8, 124.5, 124.2, 123.9, 122.6, 43.8, 29.8, 20.6, 7.5. Anal. calcd for C_18_H_16_O_2_: C, 81.74; H, 6.10. Found: C, 81.68; H, 6.02.

#### 1-(Naphthalene-yl)pentan-3-one 7o (60% yield)

NMR ^1^H (CDCl_3_, 300 MHz): *δ* 7.95 (m, 4H), 7.62 (m, 3H), 3.48 (t, *J* = 7.4 Hz, 2H), 2.84 (t, *J* = 7.6 Hz, 2H), 2.47 (q, *J* = 7.4 Hz, 2H), 1.17 (t, *J* = 7.2 Hz, 3H). NMR ^13^C (CDCl_3_, 75 MHz): *δ* 210.5, 137.1, 133.6, 131.7, 128.8, 127.7, 127.1, 125.8, 125.4, 125.3, 123.3, 42.8, 35.8, 26.6, 7.6. Anal. calcd for C_15_H_16_O: C, 84.87; H, 7.60. Found: C, 84.82; H, 7.56.

#### 4-(Naphthalene-1-yl)butan-2-one 7p (55% yield)

NMR ^1^H (CDCl_3_, 300 MHz): *δ* 7.85 (d, *J* = 7.9 Hz, 1H), 7.75 (m, 3H), 7.46 (m, 3H), 3.30 (t, *J* = 7.4 Hz, 2H), 2.79 (t, *J* = 7.2 Hz, 2H), 2.07 (s, 3H). NMR ^13^C (CDCl_3_, 75 MHz): *δ* 207.6, 136.8, 133.6, 131.5, 128.8, 127.8, 126.8, 125.9, 125.8, 125.4, 125.3, 44.2, 29.9, 26.6. Anal. calcd for C_14_H_14_O: C, 84.81; H, 7.12. Found: C, 84.75; H, 7.05.

#### 4-(Isoquinolin-4-yl)butan-2-one 7q (75% yield)

NMR ^1^H (CDCl_3,_ 300 MHz): *δ* 9.10 (s, 1H), 8.40 (s, 1H), 8.12 (d, *J* = 7.5 Hz, 1H), 7.7 (m, 3H), 3.27 (t, *J* = 7.2 Hz, 2H), 2.85 (t, *J* = 7.2 Hz, 2H), 2.16 (s, 3H). NMR ^13^C (CDCl_3_, 75 MHz): *δ* 206.8, 151.3, 144.2, 133.9, 131.2, 129.8, 128.1, 127.4, 126.6, 125.4, 42.5, 29.7, 23.1. Anal. calcd for C_13_H_13_NO: C, 78.36; H, 6.58; N, 7.03. Found: C, 78.31; H, 6.52; N, 6.97.

#### 4-(Quinolin-3-yl)butan-2-one 7r (65% yield)

NMR ^1^H (CDCl_3_, 300 MHz): *δ* 8.65 (s, 1H), 8.01 (s, 1H), 7.88 (m, 1H), 7.45 (m, 3H), 2.85 (t, *J* = 7.4 Hz, 2H), 2.65 (t, *J* = 7.0 Hz, 2H), 1.95 (s, 3H). NMR ^13^C (CDCl_3_, 75 MHz): *δ* 206.6, 150.9, 148.9, 134.1, 130.5, 129.1, 128.1, 127.5, 127.3, 126.6, 44.1, 29.7, 26.5. Anal. calcd for C_13_H_13_NO: C, 78.36; H, 6.58; N, 7.03. Found: C, 78.32; H, 6.54; N, 6.99.

#### (*E*)-5-(3-Hydroxy-3-methylbut-1-enyl)furan-2-carbaldehyde 7s (80% yield)

NMR ^1^H (CDCl_3_, 300 MHz): *δ* 9.45 (s, 1H), 7.17 (d, *J* = 3.6 Hz, 1H), 6.64 (d, *J* = 15.8 Hz, 1H), 6.45 (d, *J* = 15.8, 1H) 6.34 (d, *J* = 3.6 Hz, 1H), 1.34 (s, 6H). NMR ^13^C (CDCl_3_, 75 MHz): *δ* 176.9, 158.3, 151.2, 143.1, 124.1, 113.8, 109.9, 70.7, 29.5. Anal. calcd for C_10_H_12_O_3_: C, 66.65; H, 6.71. Found: C, 66.59; H, 6.68.

#### 2-[(*E*)-1-Buten-3-methyl-3-ol]benzonitrile 7t (70% yield)

NMR ^1^H (CDCl_3_, 300 MHz): *δ* 7.45 (m, 3H), 7.16 (dd, *J* = 8.0, 7.8 Hz, 2H), 6.79 (d, *J* = 16 Hz, 1H), 6.43 (d, *J* = 16 Hz, 1H), 1.34 (s, 6H). NMR ^13^C (CDCl_3_, 75 MHz): *δ* 142.9, 140.3, 132.7, 132.6, 127.3, 125.7, 122.2, 117.8, 110.6, 70.9, 29.4. Anal. calcd for C_12_H_13_NO: C, 76.98; H, 7.00; N, 7.48. Found: C, 76.94; H, 6.95; N, 7.43.

#### (*E*)-2-Metil-4-(quinolin-3-il)-3-buten-2-ol 7u (85% yield)

NMR ^1^H (CDCl_3_, 300 MHz): *δ* 8.64 (brs, 1H), 8.04 (brs, 1H), 7.9 (d, *J* = 8.0 Hz, 1H), 7.43 (m, 2H), 7.36 (m, 1H), 6.61 (d, *J* = 16.2 Hz, 1H), 6.44 (d, *J* = 16.2, 1H), 1.33 (s, 6H). NMR ^13^C (CDCl_3_, 75 MHz): *δ* 149.1, 145.6, 136.9, 129.4, 128.8, 127.3, 126.6, 140.4, 122.7, 116.7, 70.3, 29.7. Anal. calcd for C_14_H_15_NO: C, 78.84; H, 7.09; N, 6.57. Found: C, 78.79; H, 7.02; N, 6.51.

#### (3*E*,*E*′)-4,4′-Pyridine-3,5-di(yl)bis(3-buten-2-methyl-2-ol) 7v (75% yield)

NMR ^1^H (CDCl_3,_ 300 MHz): *δ* 8.56 (brs, 2H), 7.98 (brs, 1H), 6.51 (d, *J* = 15.9 Hz, 2H), 6.38 (d, *J* = 15.9, 2H), 1.39 (s, 12H). NMRN ^13^C (CDCl_3_, 75 MHz): *δ* 149.0, 141.7, 135.3, 121.4, 120.8, 70.9, 29.8. Anal. calcd for C_15_H_21_NO_2_: C, 72.84; H, 8.56; N, 5.66. Found: C, 72.80; H, 8.51; N, 5.62.

## Conflicts of interest

There are no conflicts to declare.

## Supplementary Material

RA-011-D1RA03484G-s001
