# Supplementary material for: The Heck reaction of allylic alcohols catalysed by an N-heterocyclic carbene-Pd(ii) complex and toxicity of the ligand precursor for the marine benthic copepod Amphiascoides atopus
Source: RSC Adv. 2021 Jun 7;11(33):20278–84. doi: 10.1039/d1ra03484g (PMC9033956; doi:10.1039/d1ra03484g)
Supplement: RA-011-D1RA03484G-s001 [file RA-011-D1RA03484G-s001.pdf]

Electronic Supplementary Material (ESI) for RSC Advances.
This journal is © The Royal Society of Chemistry 2021

Supporting Information

# The Heck reaction of allylic alcohols catalysed by an N-heterocyclic carbene-Pd(II) complex and toxicity of the ligand precursor for the marine benthic copepod *Amphiascoides atopus*

Jorge Cárdenas,[a] Rubén Gaviño,[a] Eréndira García-Ríos,[a] Lucero Rios-Ruiz,[a] Ana C. Puello-Cruz,[b] Francisco Neptalí Morales-Serna,*[c] Samuel Gómez,[c] Adolfo López-Torres,[d] José Antonio Morales-Serna*[d]

[a]*Instituto de Química, Universidad Nacional Autónoma de México, Circuito Exterior, Ciudad Universitaria, Ciudad de México, 04510, México.*
[b]*Instituto de Ciencias del Mar y Limnología, Universidad Nacional Autónoma de México, Mazatlán, Sinaloa, 82040, México.*
[c]*Centro de Investigación en Alimentación y Desarrollo, A.C. Unidad Mazatlán en Acuicultura y Manejo Ambiental, Mazatlán, Sinaloa, 82112, México.*
[d]*Instituto de Química Aplicada, Universidad del Papaloapan, Tuxtepec, Oaxaca, 68301, México. E-mail: joseantonio.moralesserna@gmail.com*

**Table of Contents**

1. NMR spectra S1

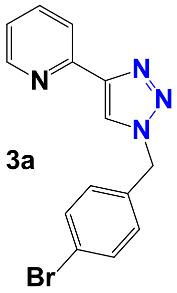

**3a**

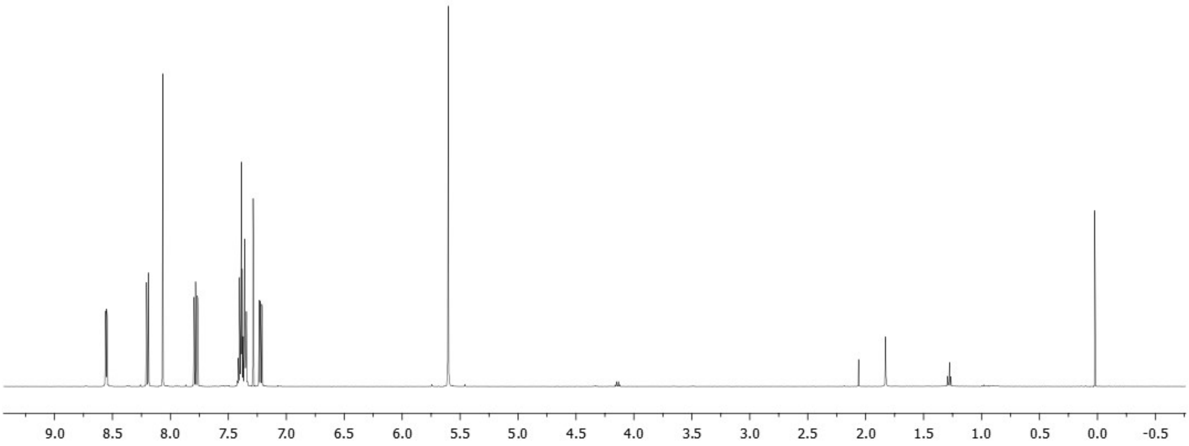

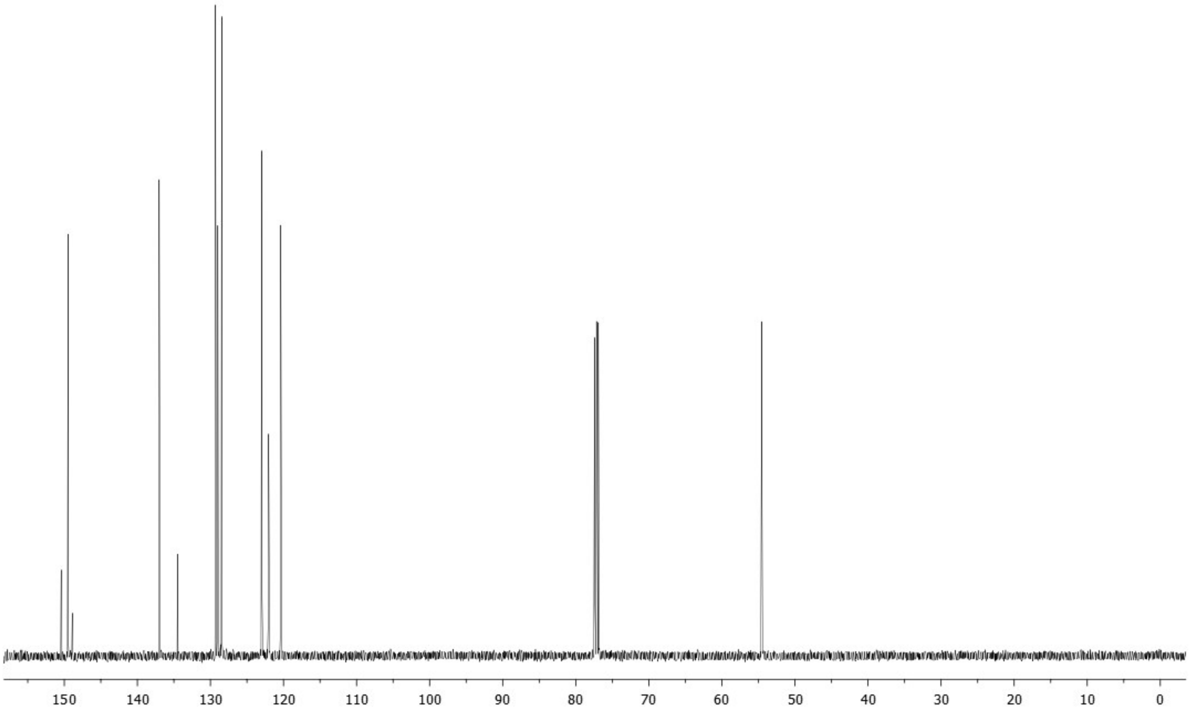

S1. $^{1}H$ NMR and $^{13}C$ NMR in $CDCl_3$ of **3a**

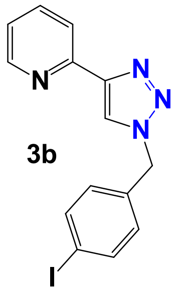


3b

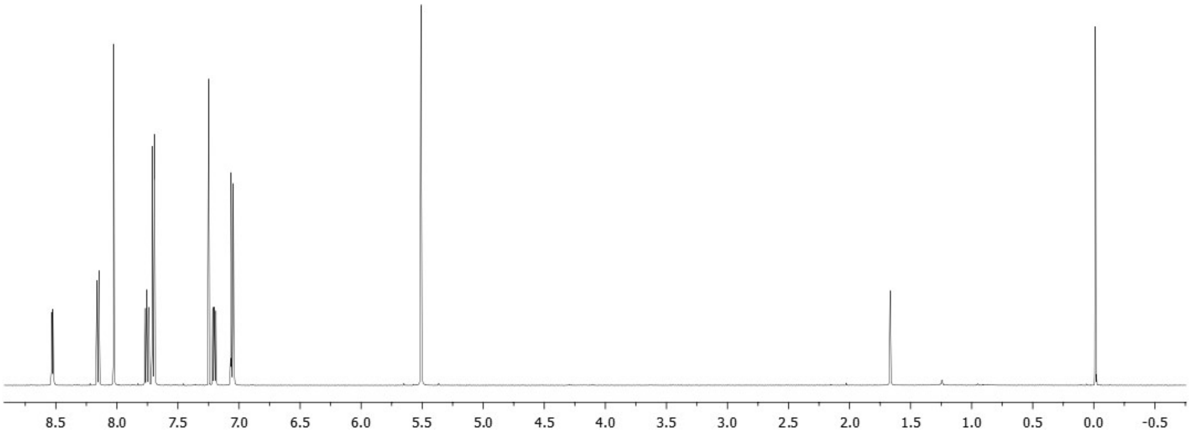


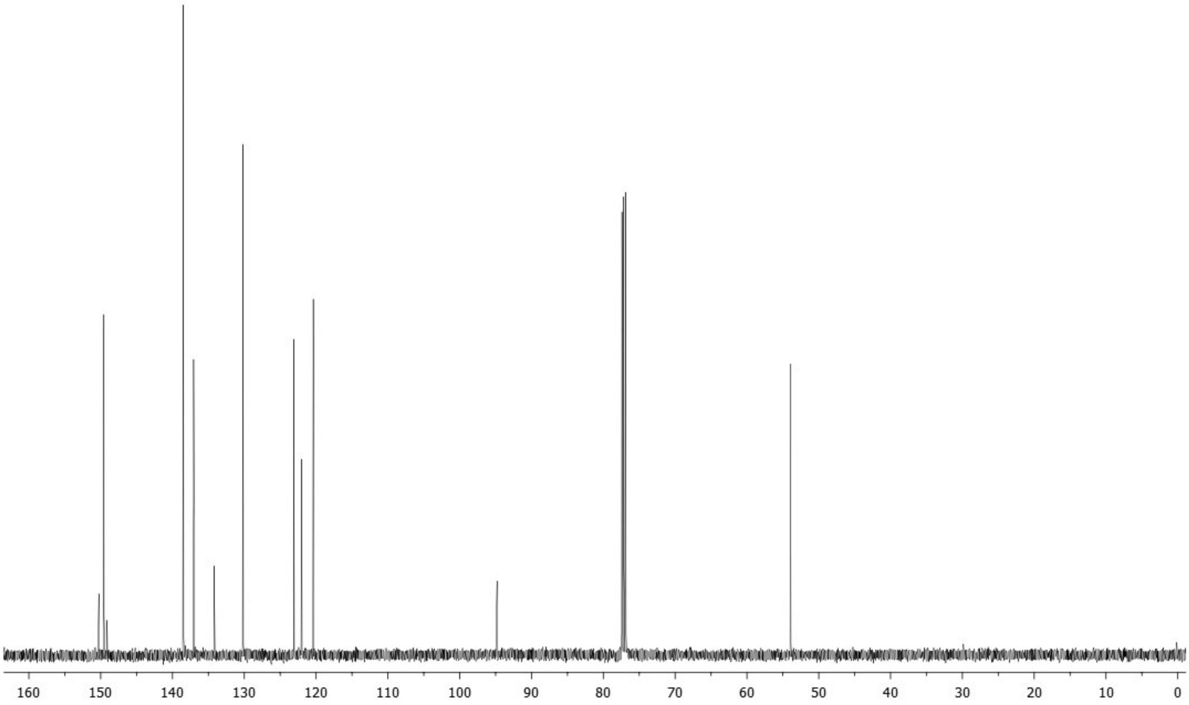


S2. $^{1}H$ NMR and $^{13}C$ NMR in $CDCl_3$ of **3b**

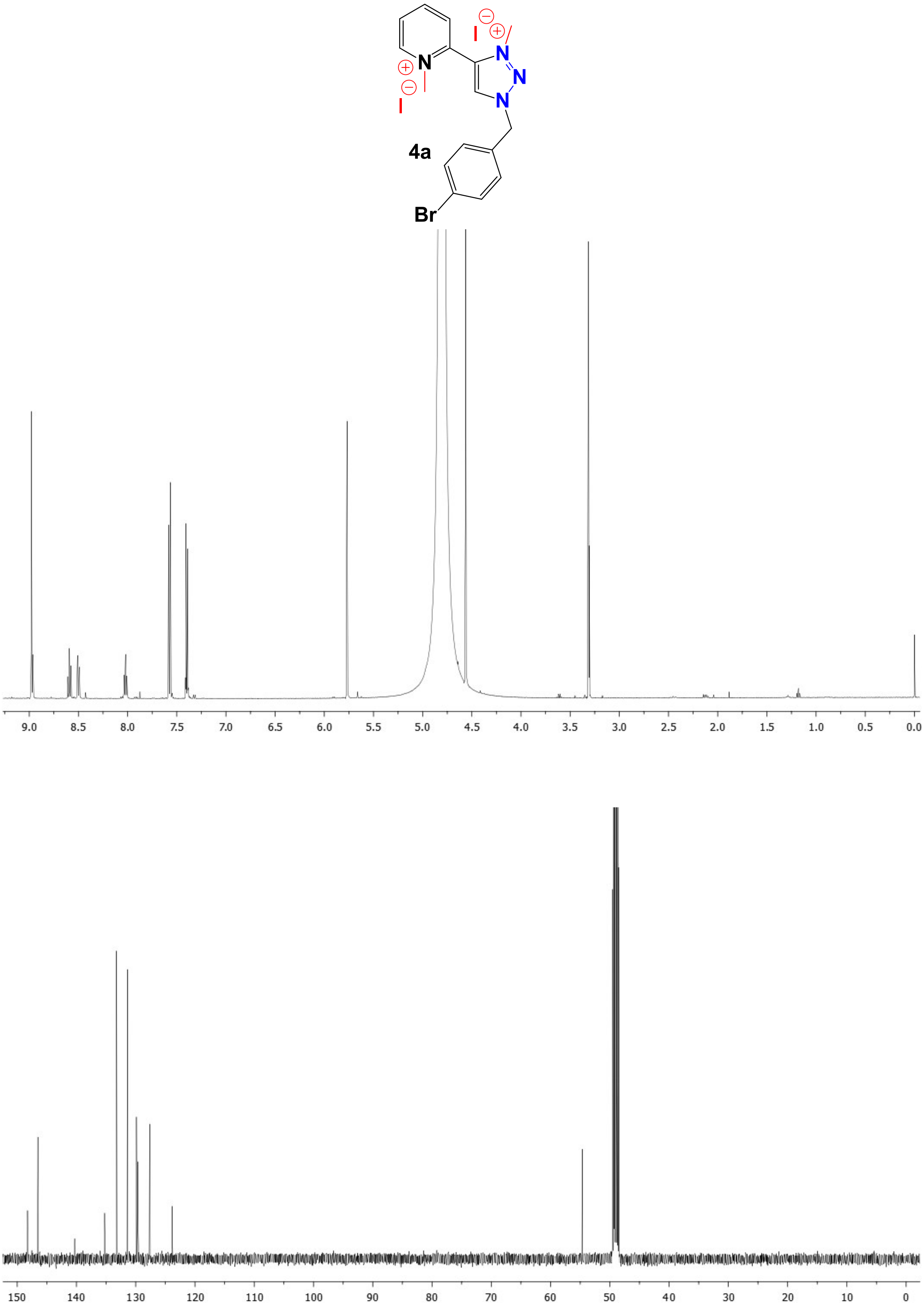


S3. $^{1}H$ NMR and $^{13}C$ NMR in $CDCl_3$ of **4a**

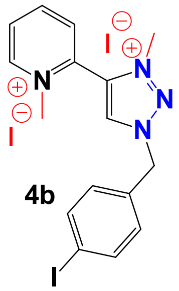


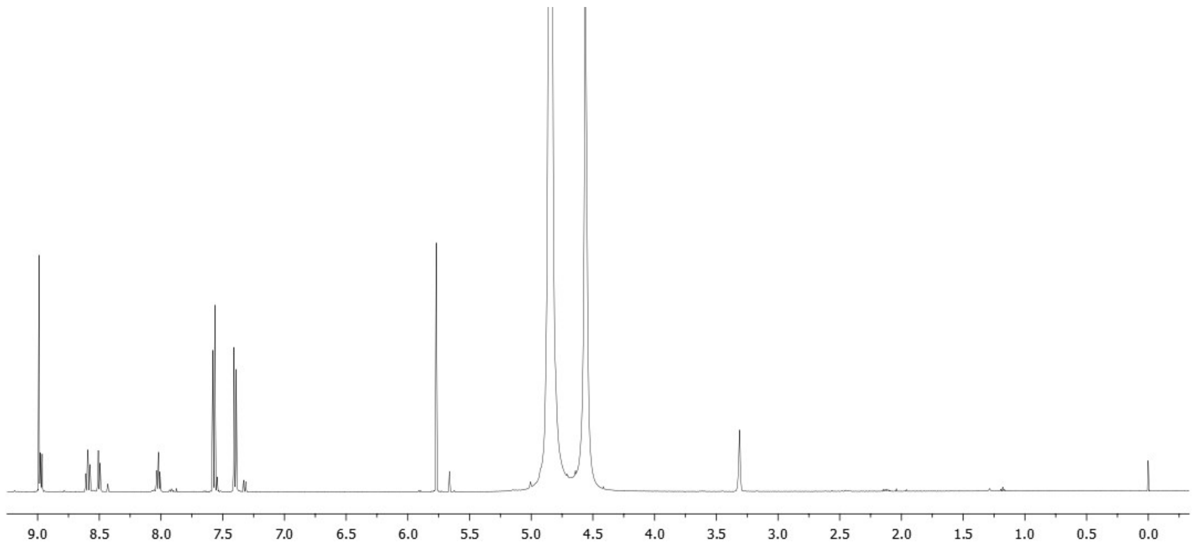


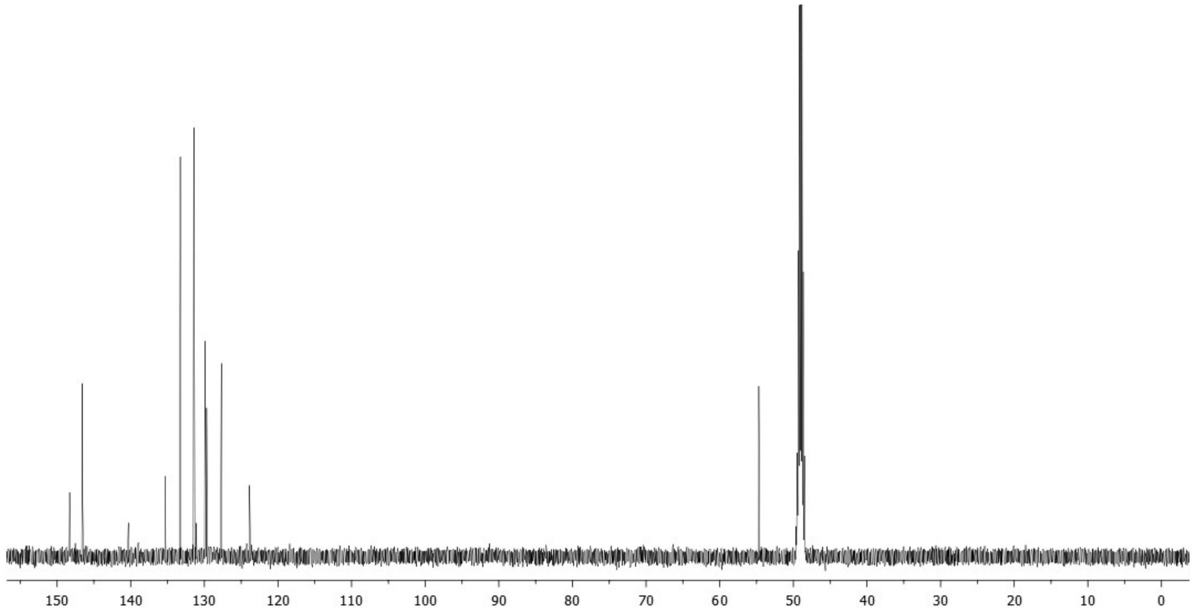


S4. $^{1}H$ NMR and $^{13}C$ NMR in $CDCl_3$ of **4b**

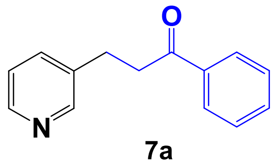


**7a**

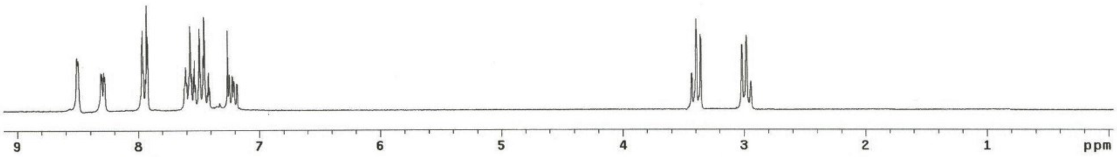


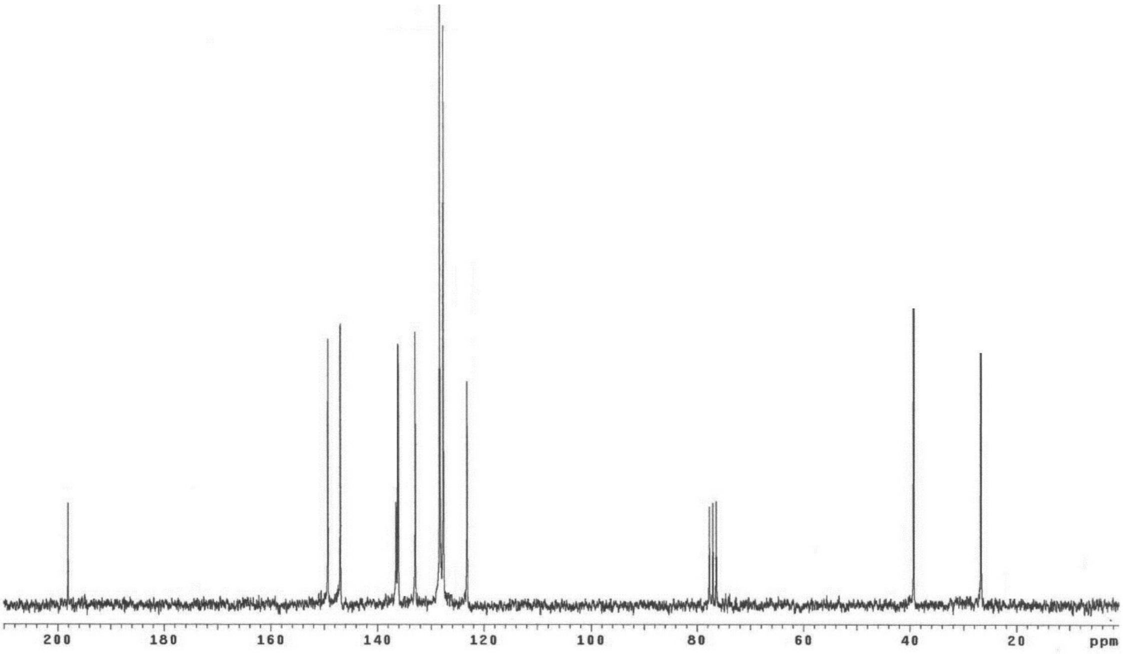


S5. $^{1}H$ NMR and $^{13}C$ NMR in $CDCl_3$ of **7a**

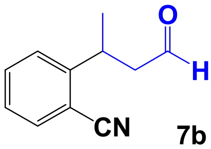


**7b**

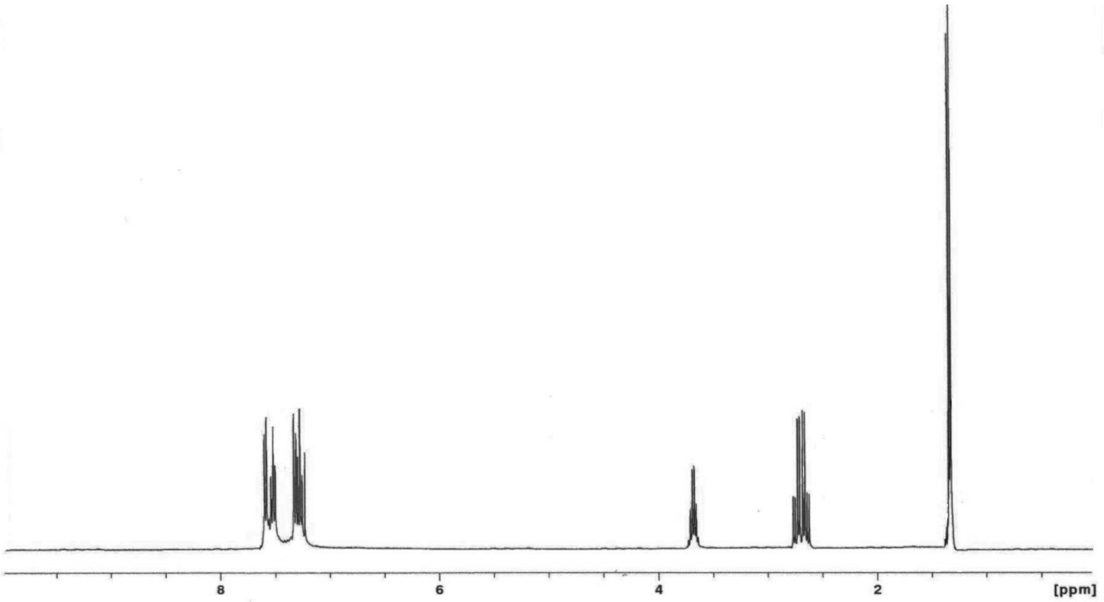


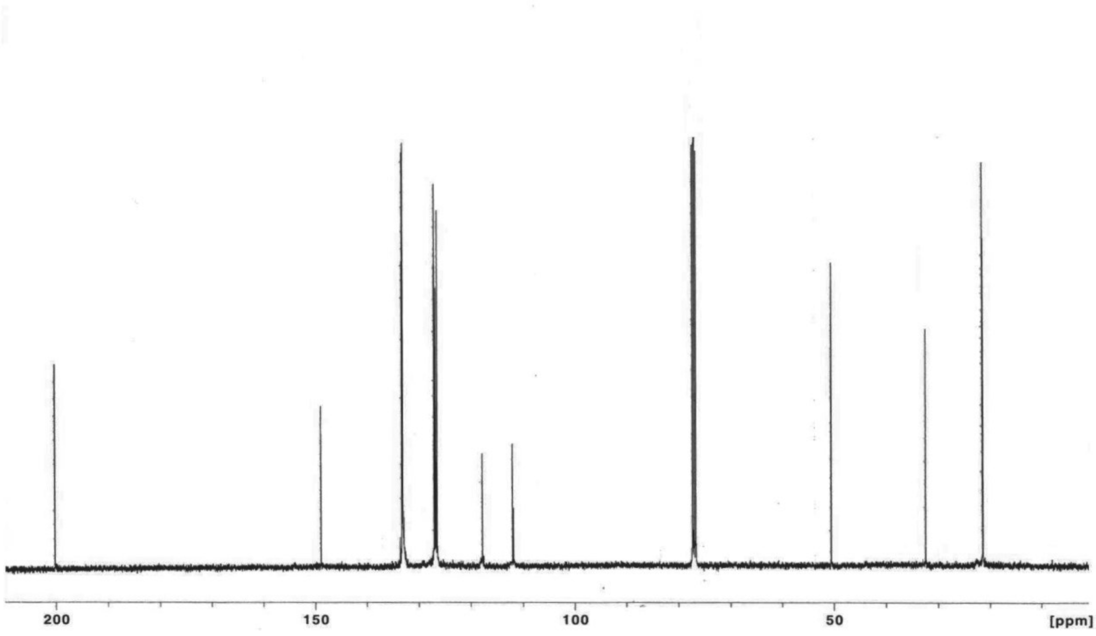


S6. $^{1}H$ NMR and $^{13}C$ NMR in $CDCl_3$ of **7b**

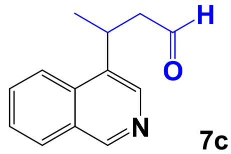


**7c**

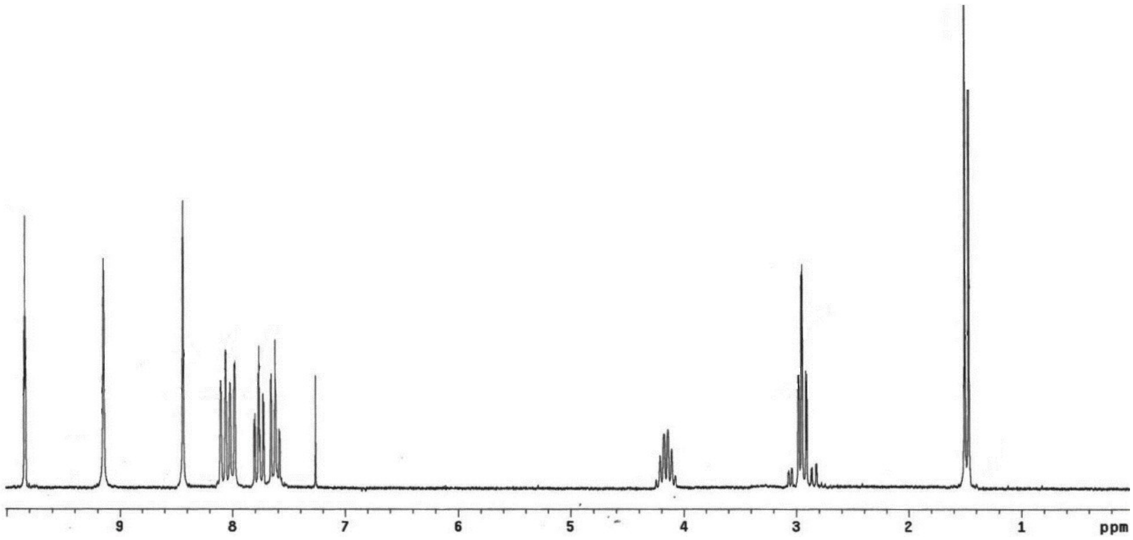


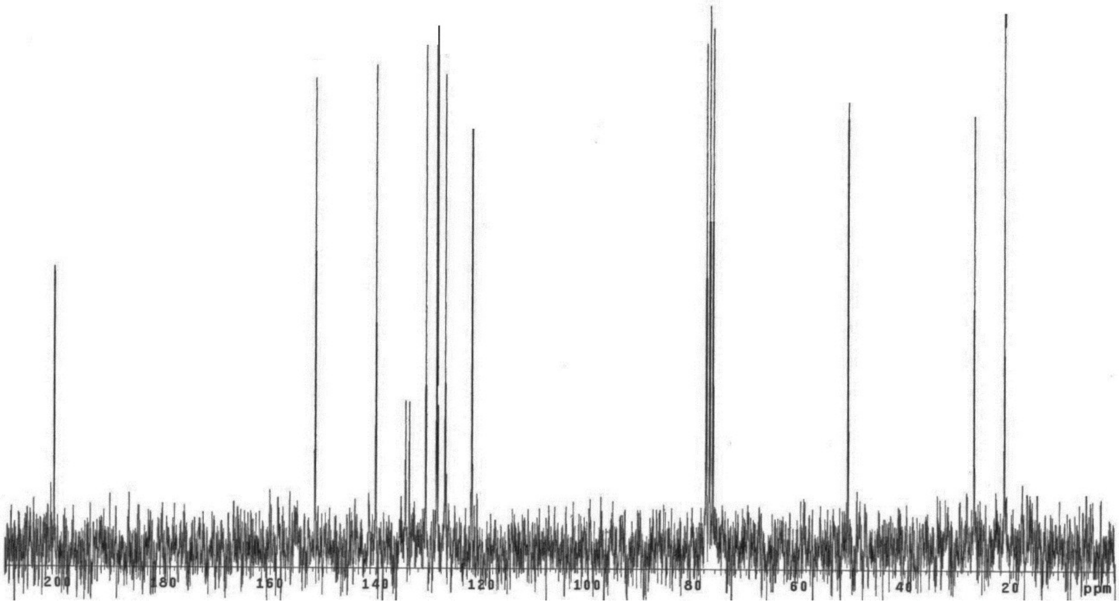


S7. $^{1}$H NMR and $^{13}$C NMR in $CDCl_3$ of **7c**

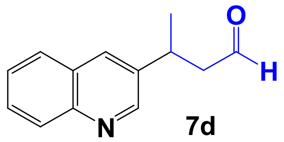

**7d**

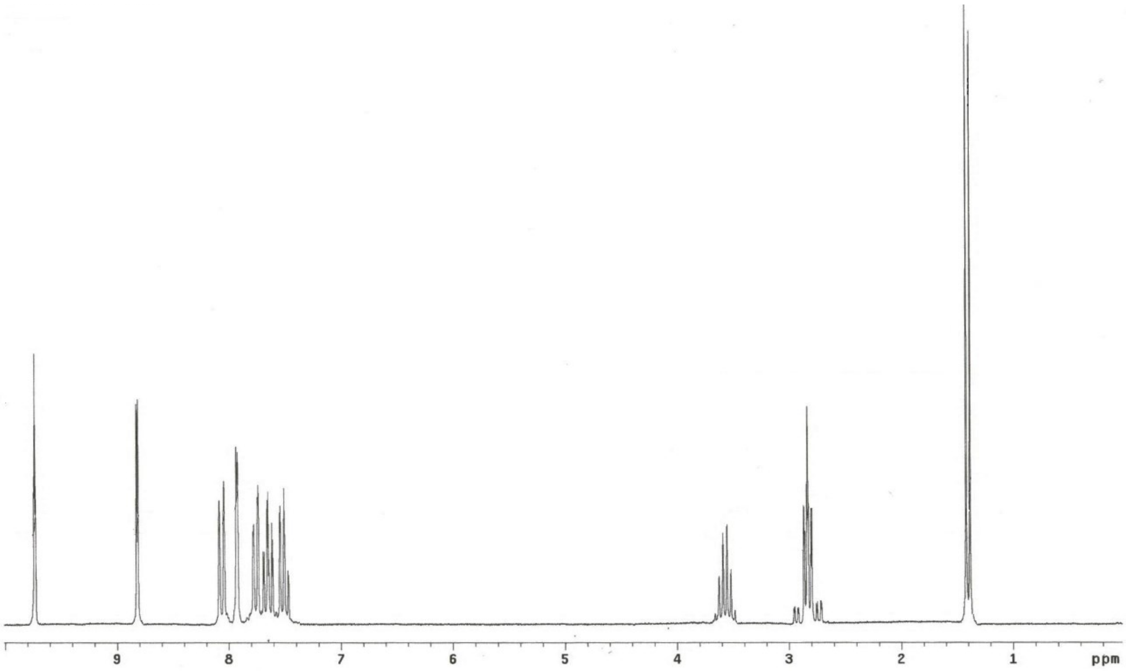


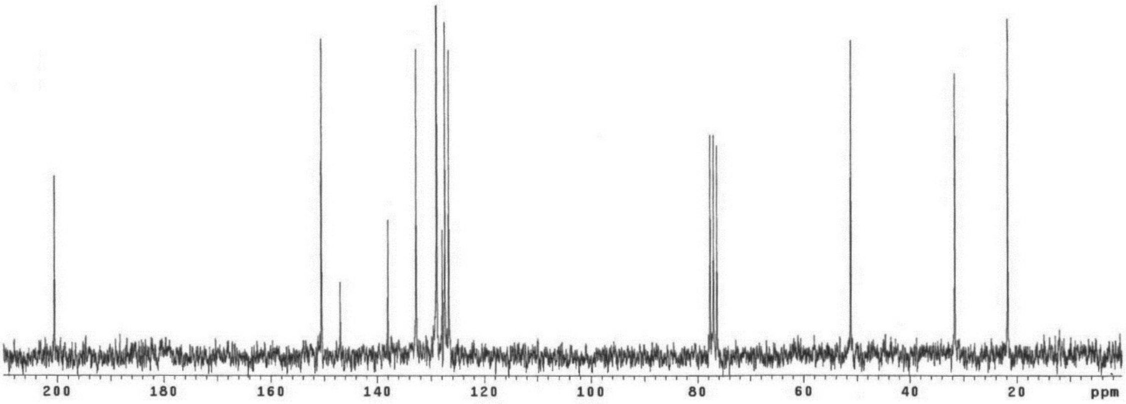


S8. $^{1}$H NMR and $^{13}$C NMR in $CDCl_3$ of **7d**

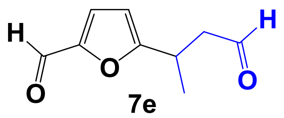


7e

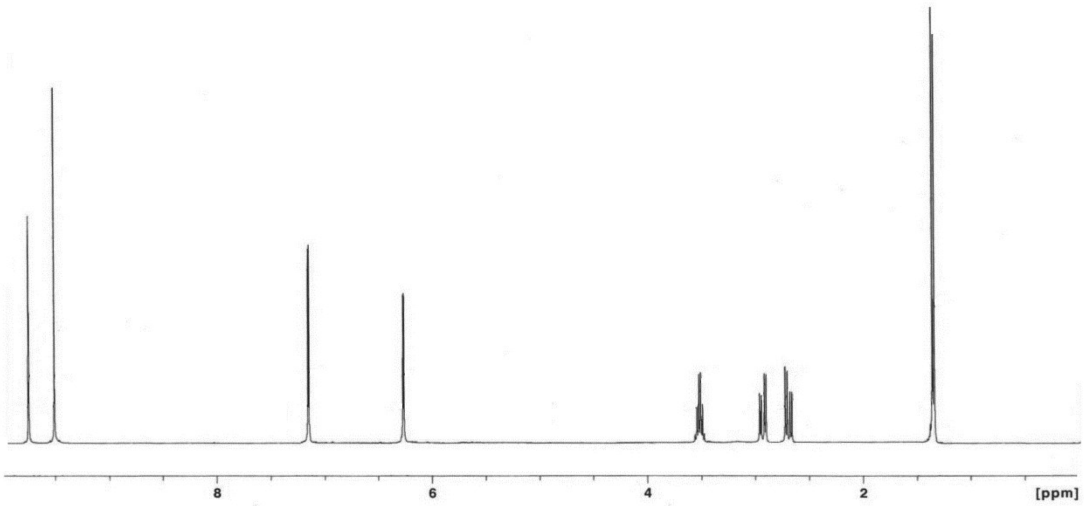


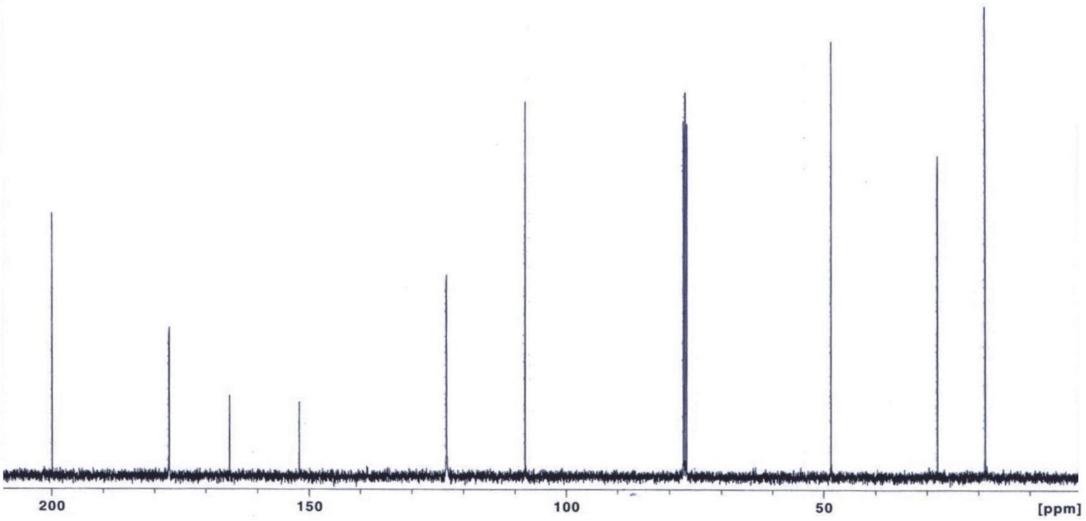


S9. $^{1}H$ NMR and $^{13}C$ NMR in $CDCl_3$ of **7e**

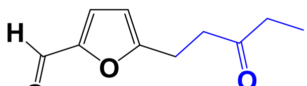

**7f**

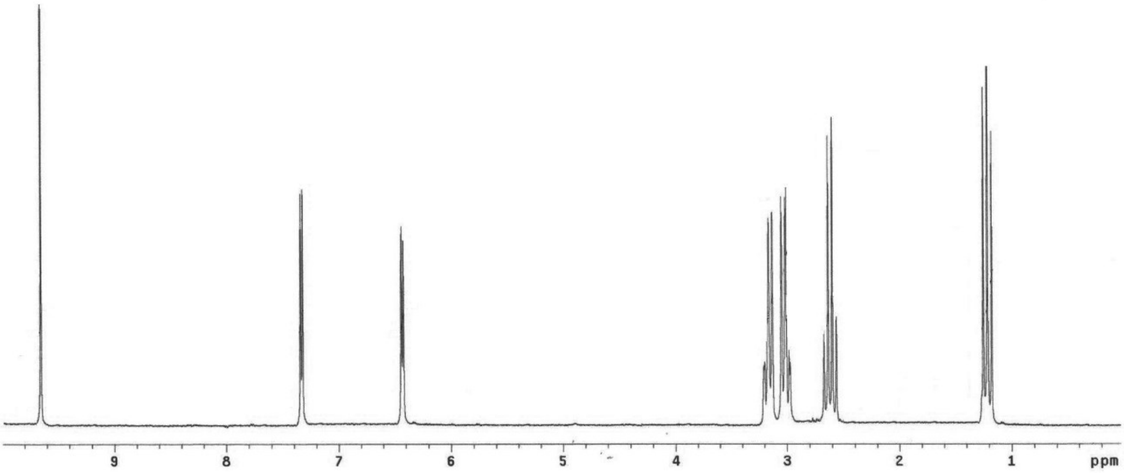


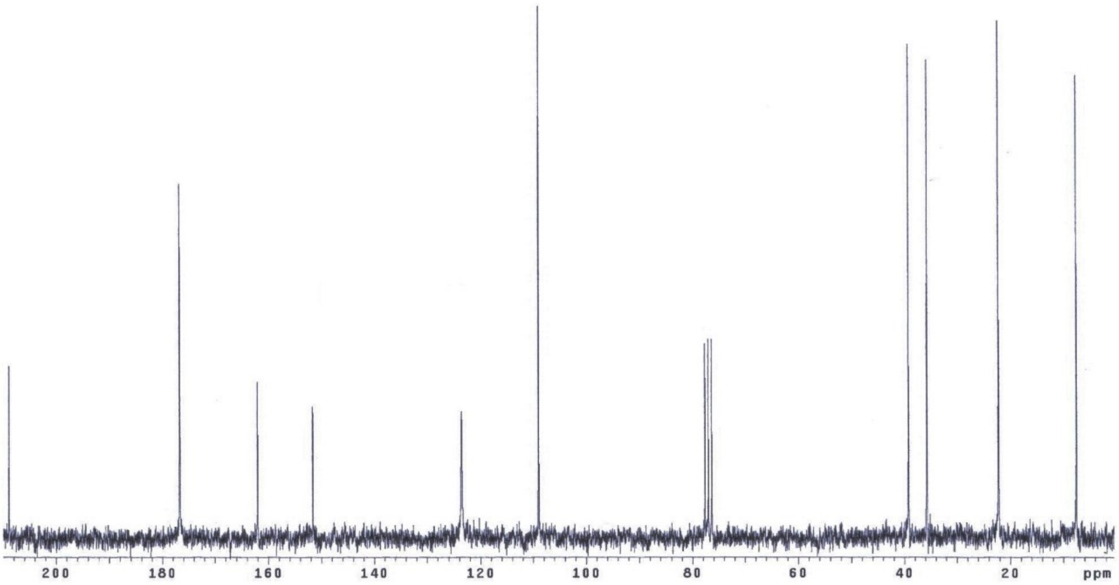


S10. $^{1}H$ NMR and $^{13}C$ NMR in $CDCl_3$ of **7f**

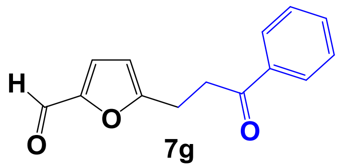

7g

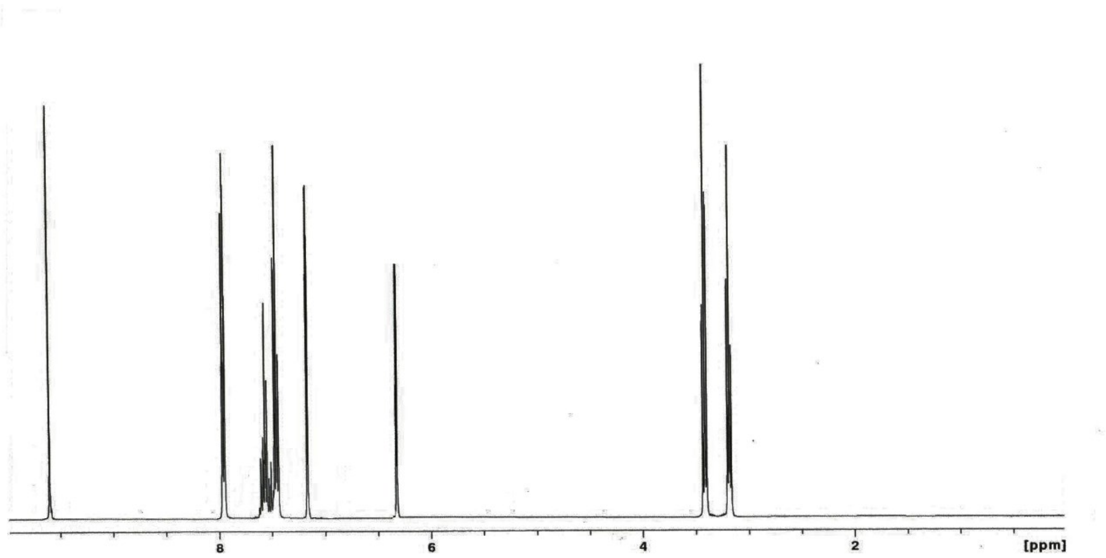

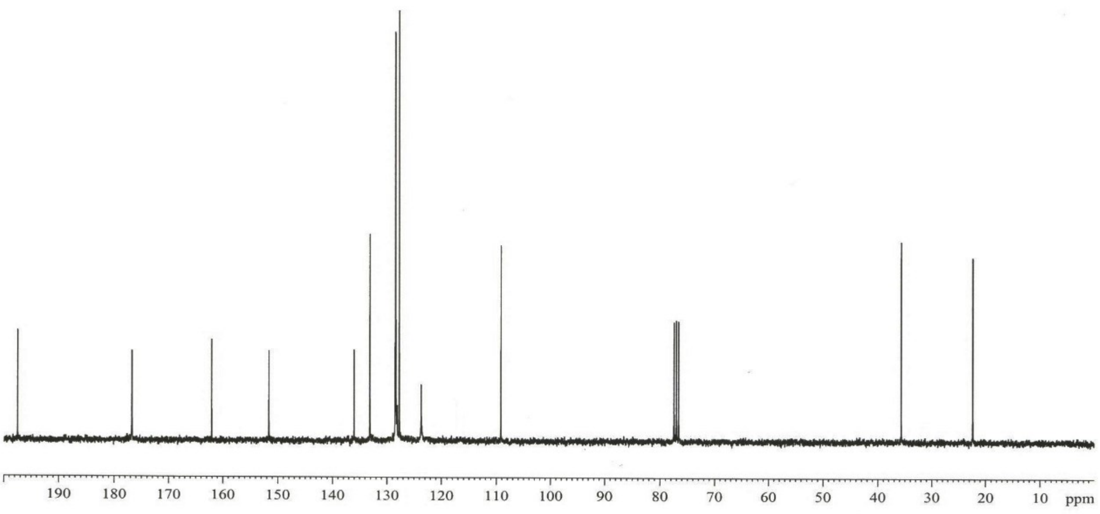

S11. $^{1}H$ NMR and $^{13}C$ NMR in $CDCl_3$ of **7g**

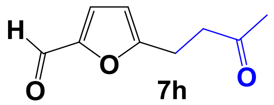

**7h**

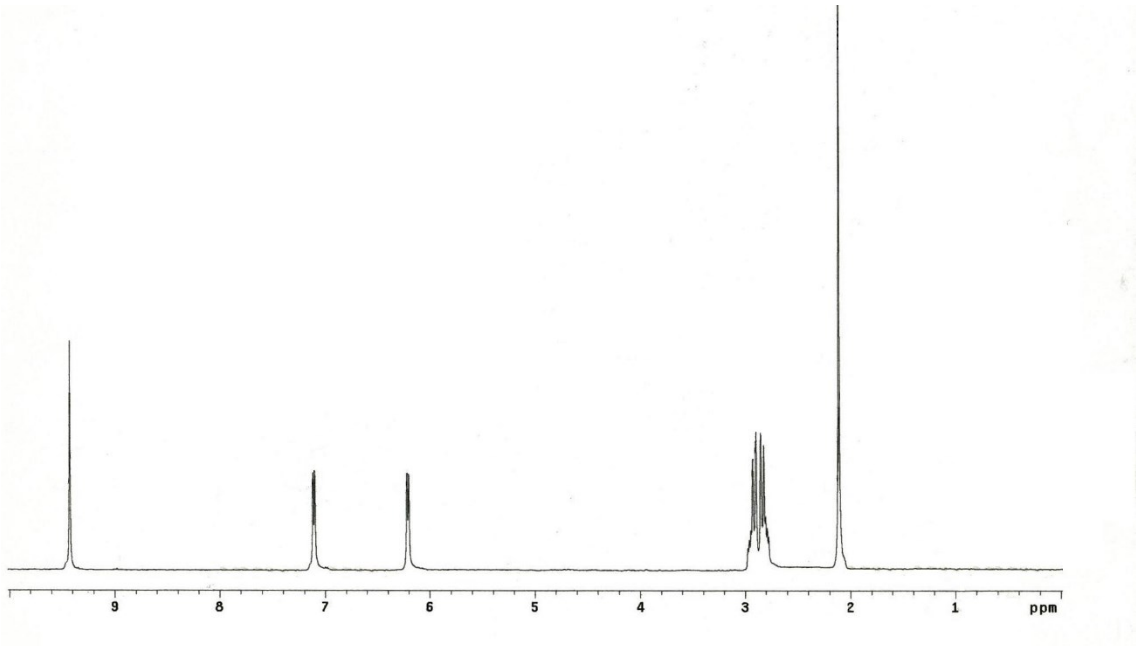


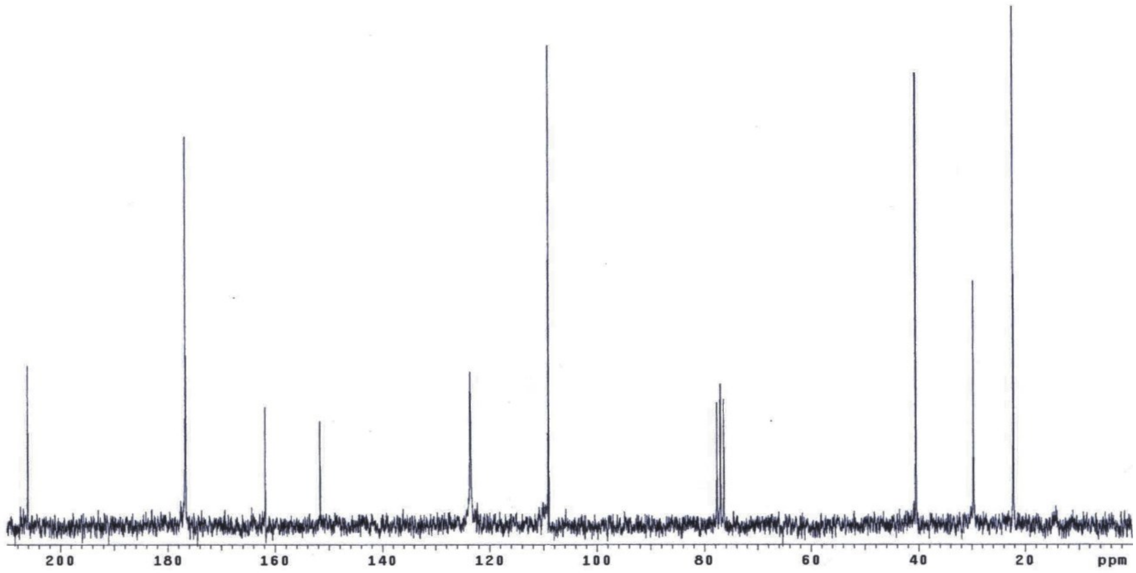


S12. $^{1}$H NMR and $^{13}$C NMR in $CDCl_3$ of **7h**

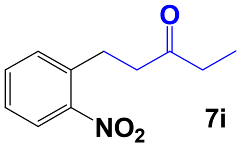

7i

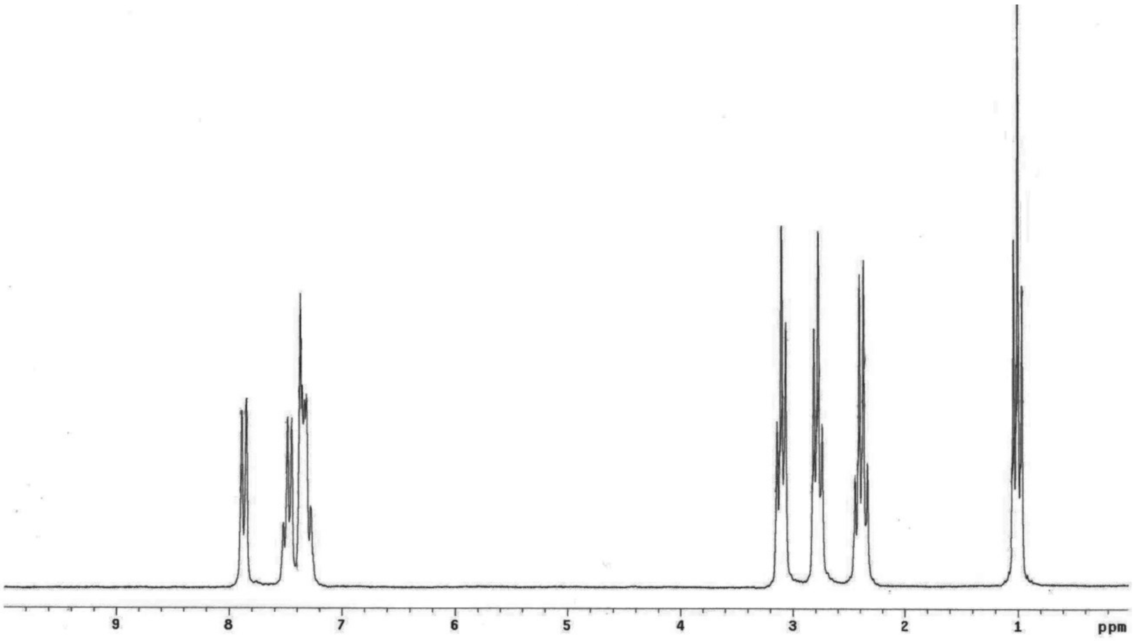

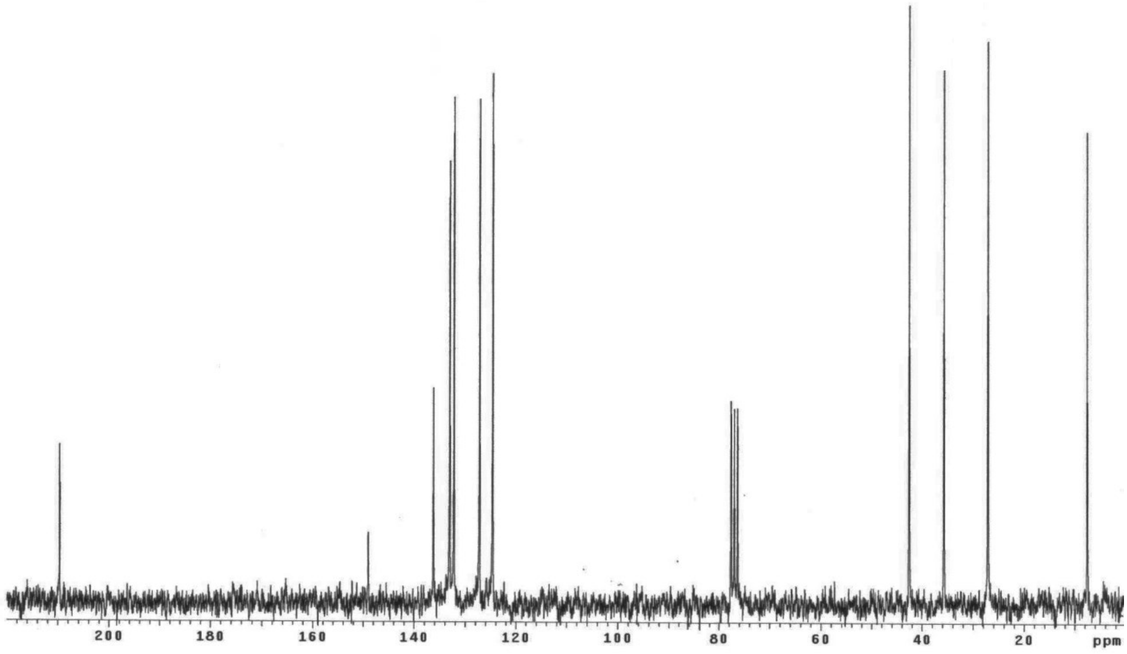

S13. $^{1}H$ NMR and $^{13}C$ NMR in $CDCl_3$ of **7i**

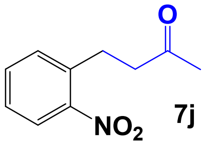


**7j**

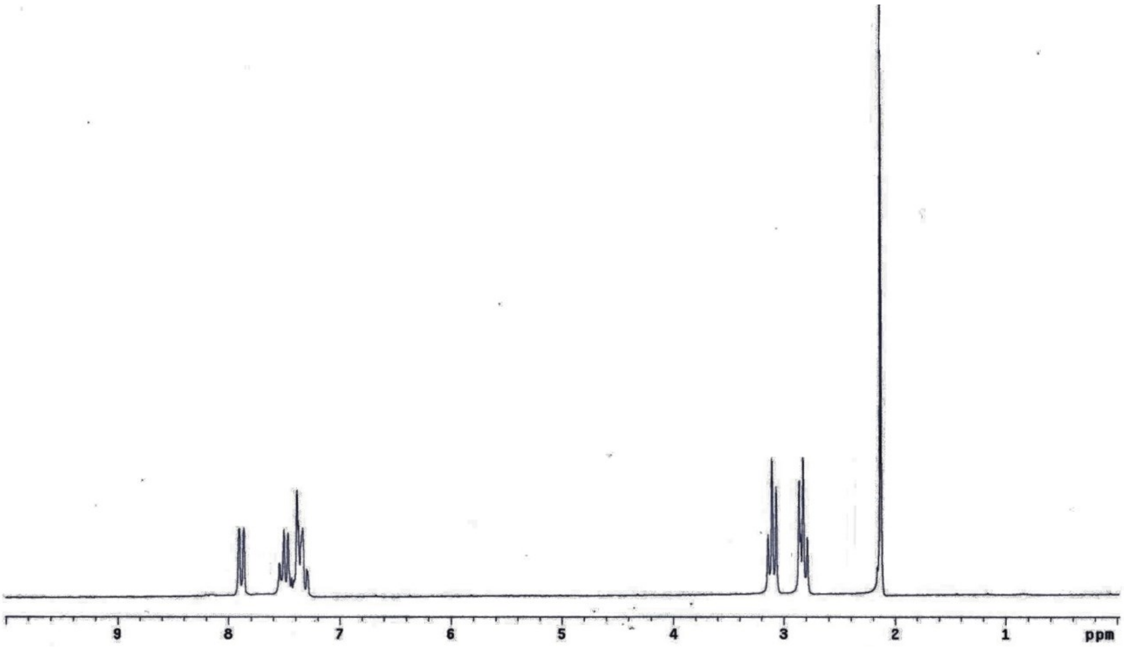


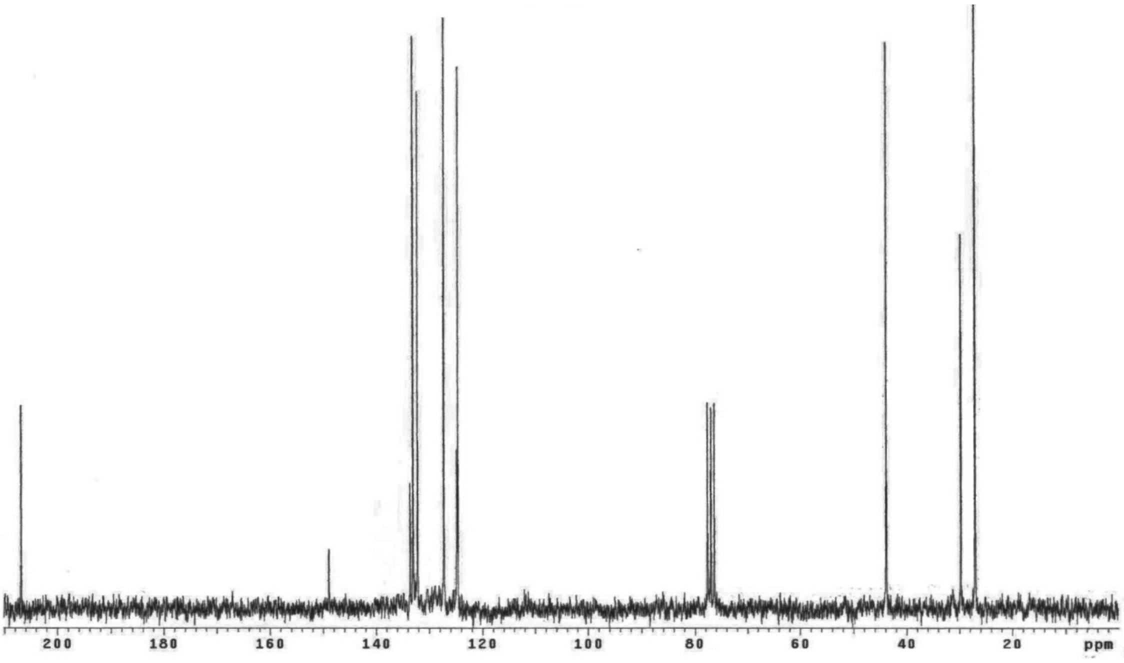


S14. $^{1}$H NMR and $^{13}$C NMR in $CDCl_3$ of **7j**

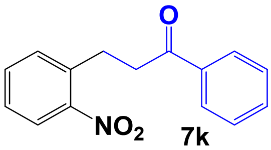


**7k**

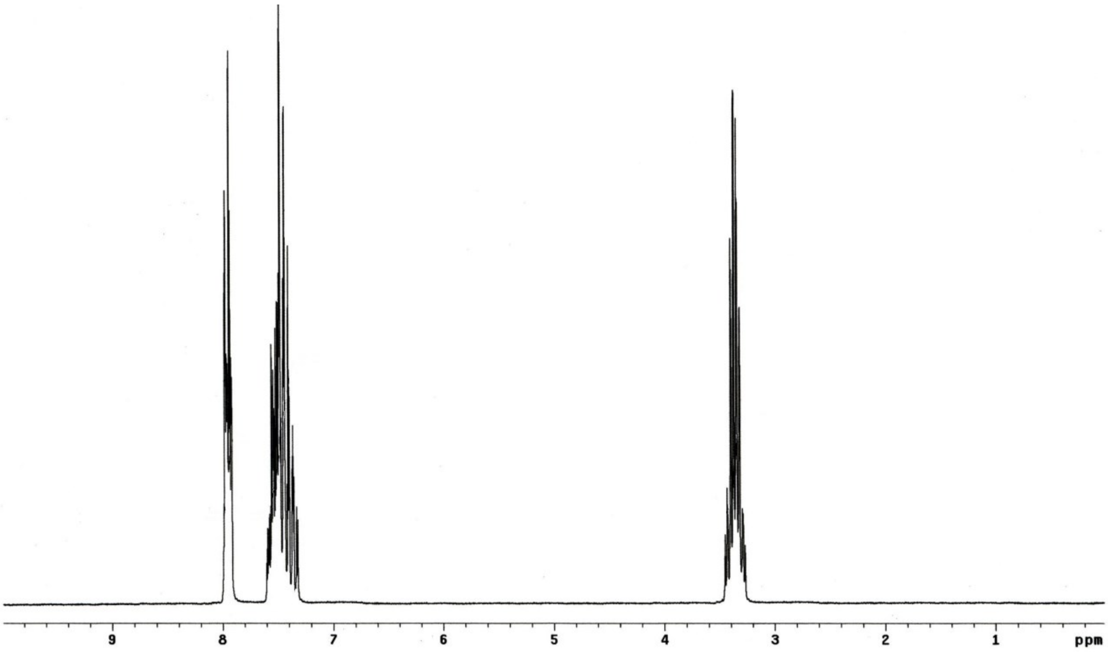

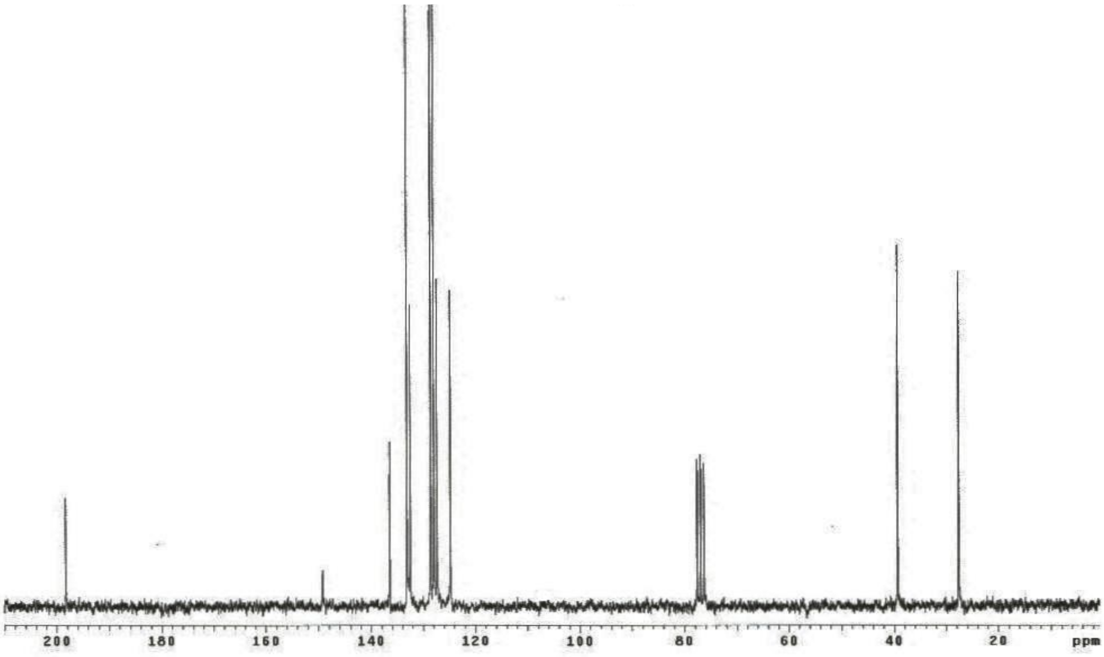

S15. $^{1}H$ NMR and $^{13}C$ NMR in $CDCl_3$ of **7k**

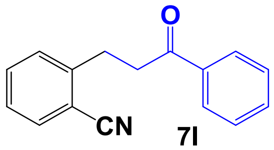


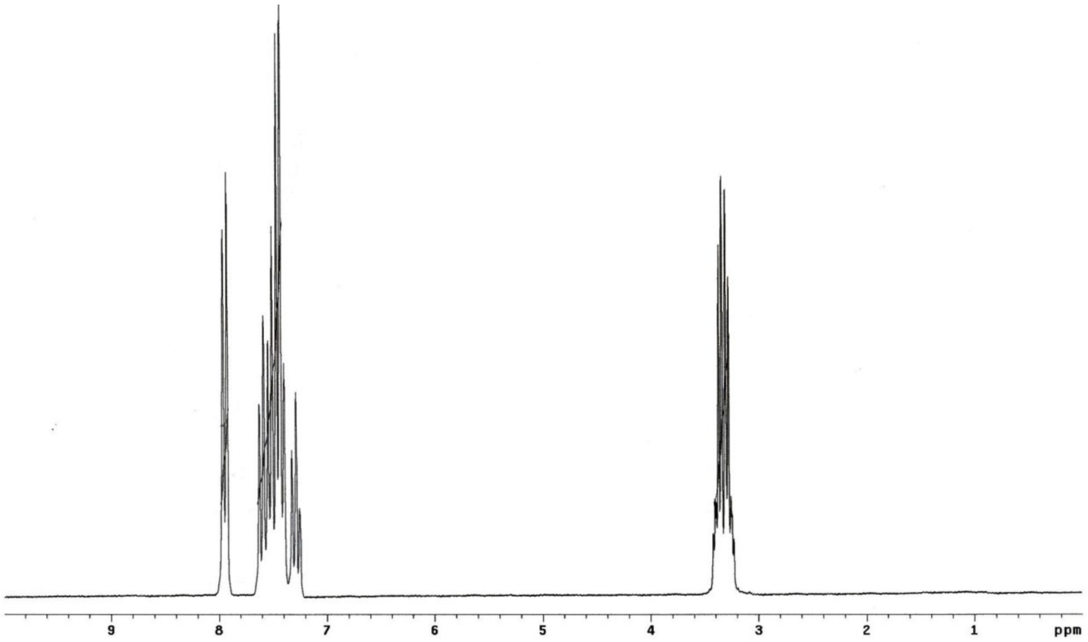


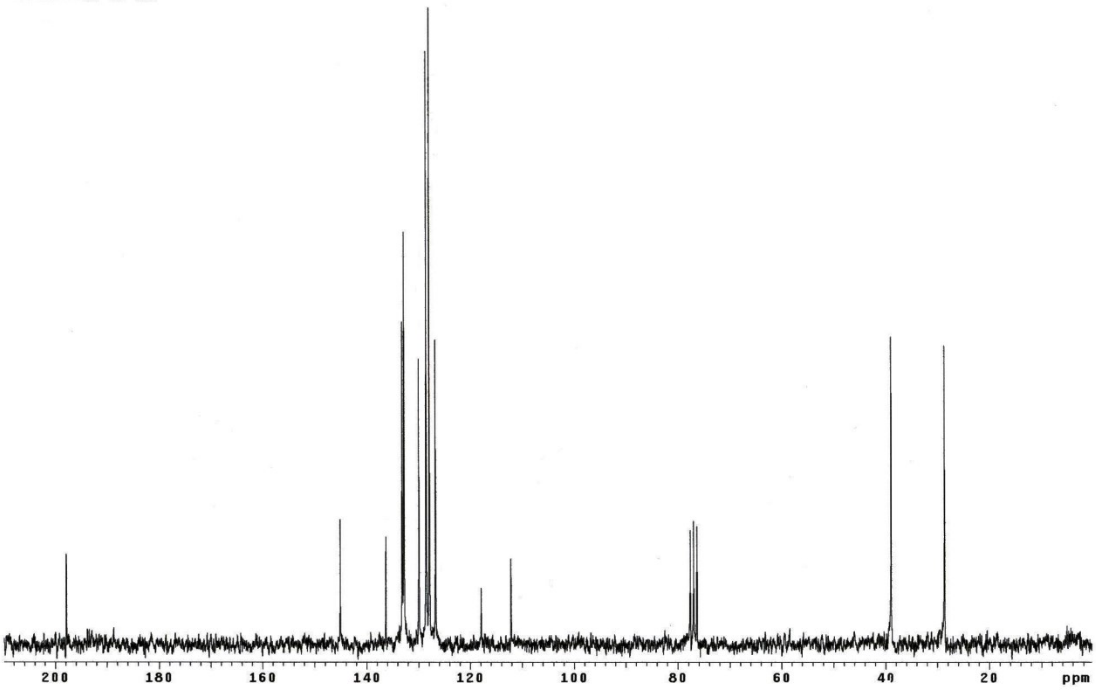


S16. $^{1}H$ NMR and $^{13}C$ NMR in $CDCl_3$ of **7l**

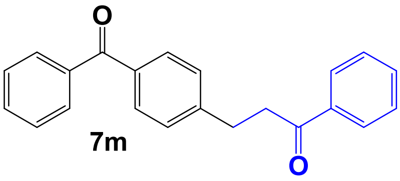

**7m**

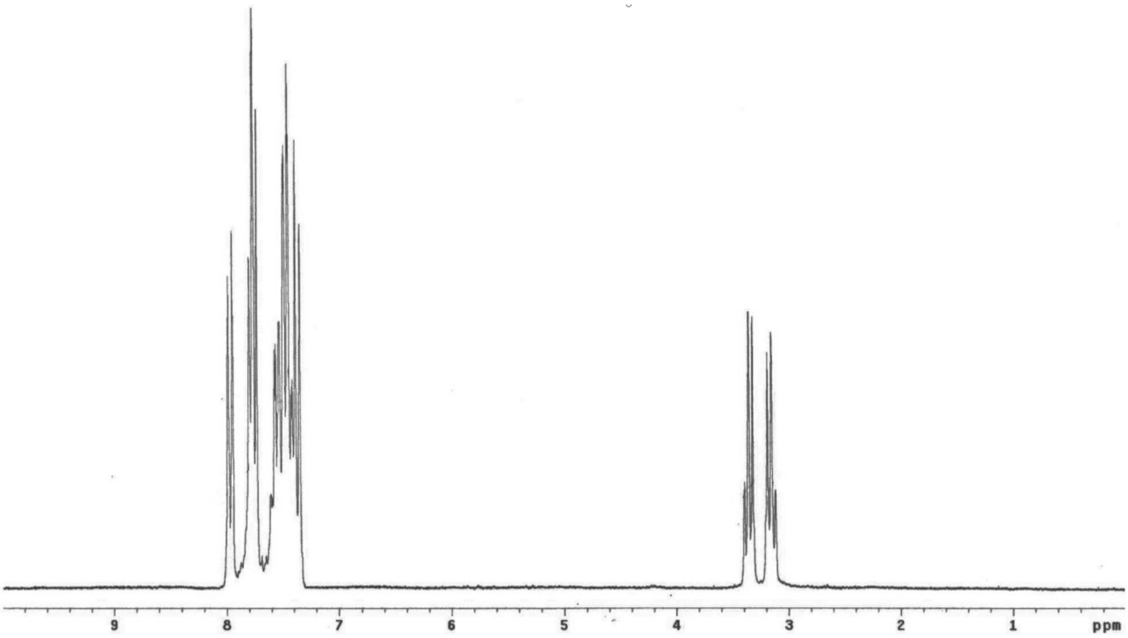

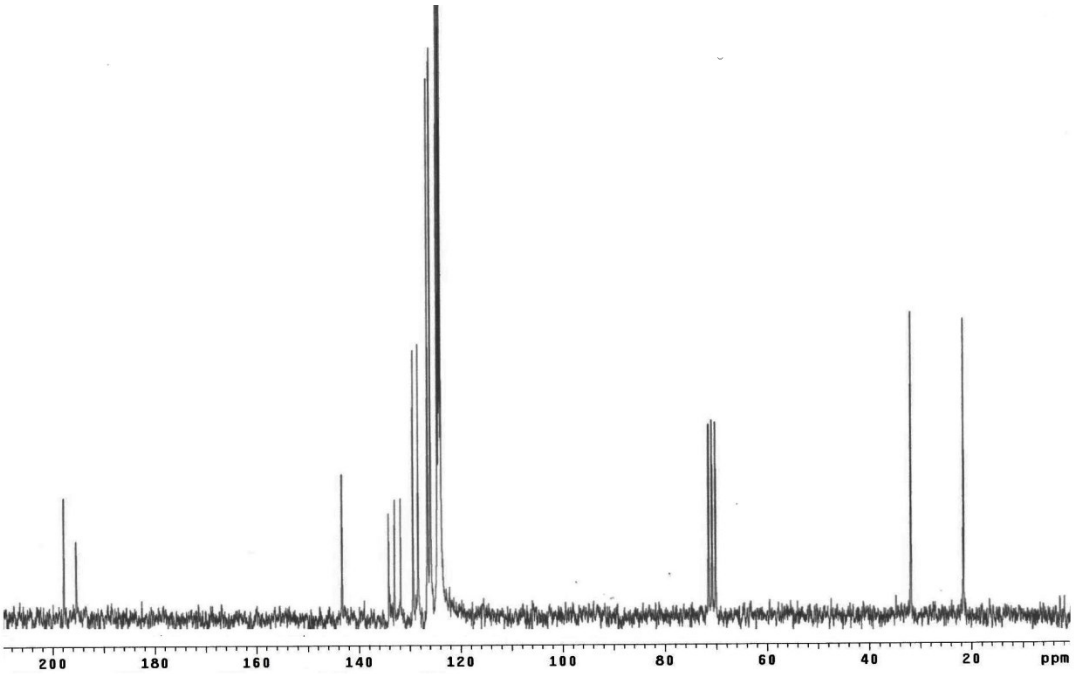

S17. $^{1}H$ NMR and $^{13}C$ NMR in $CDCl_3$ of **7m**

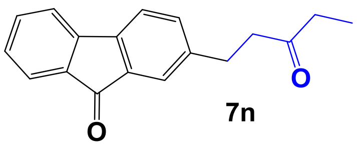


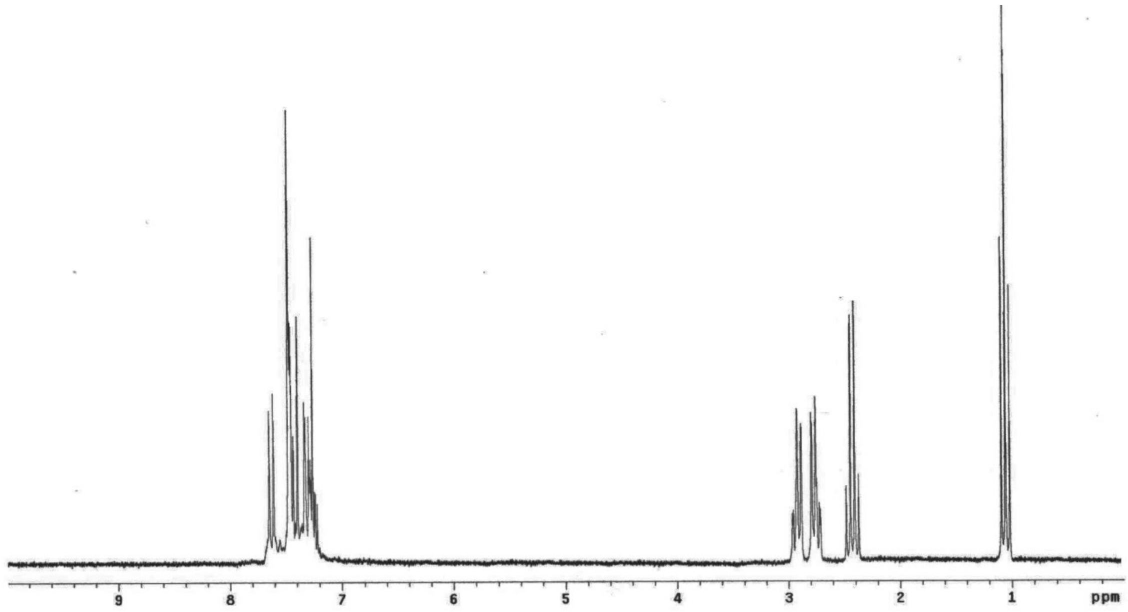


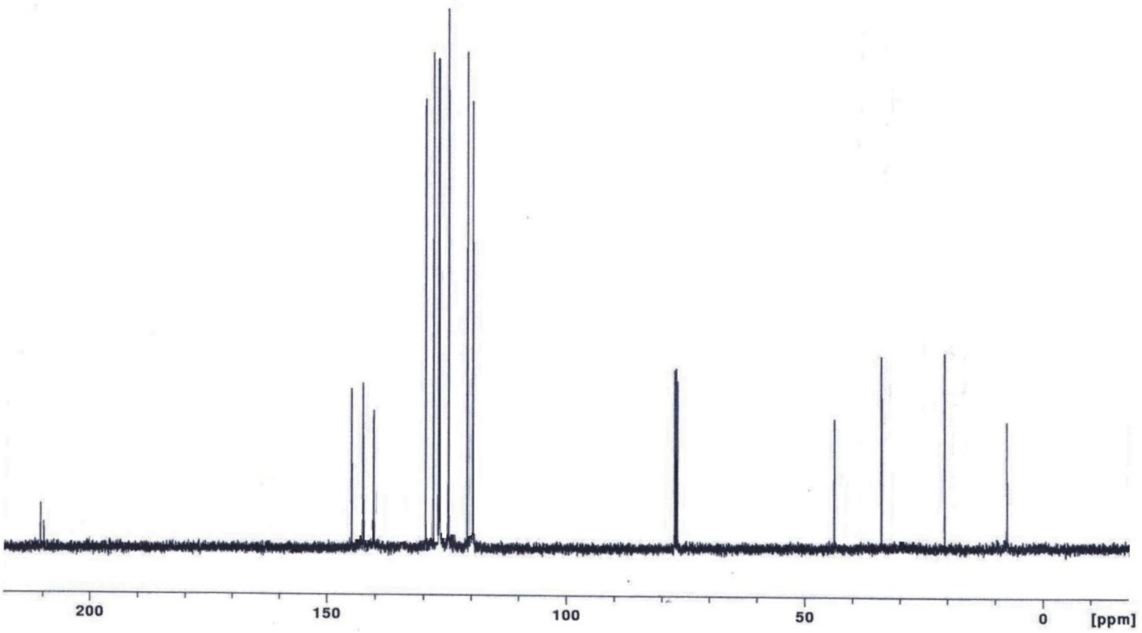


S18. $^{1}$H NMR and $^{13}$C NMR in $CDCl_3$ of **7n**

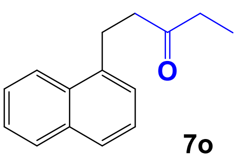

**7o**

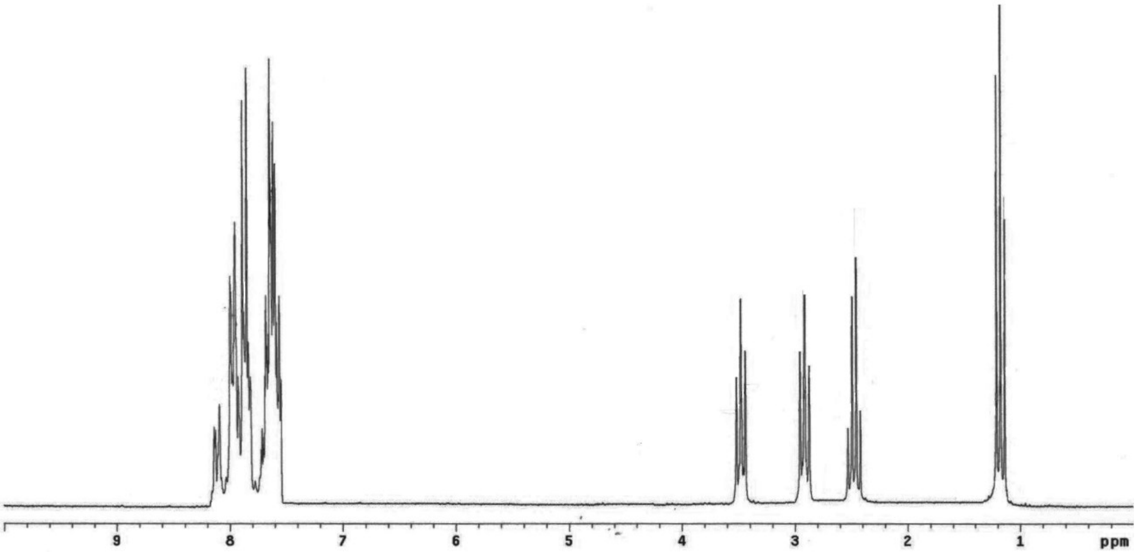


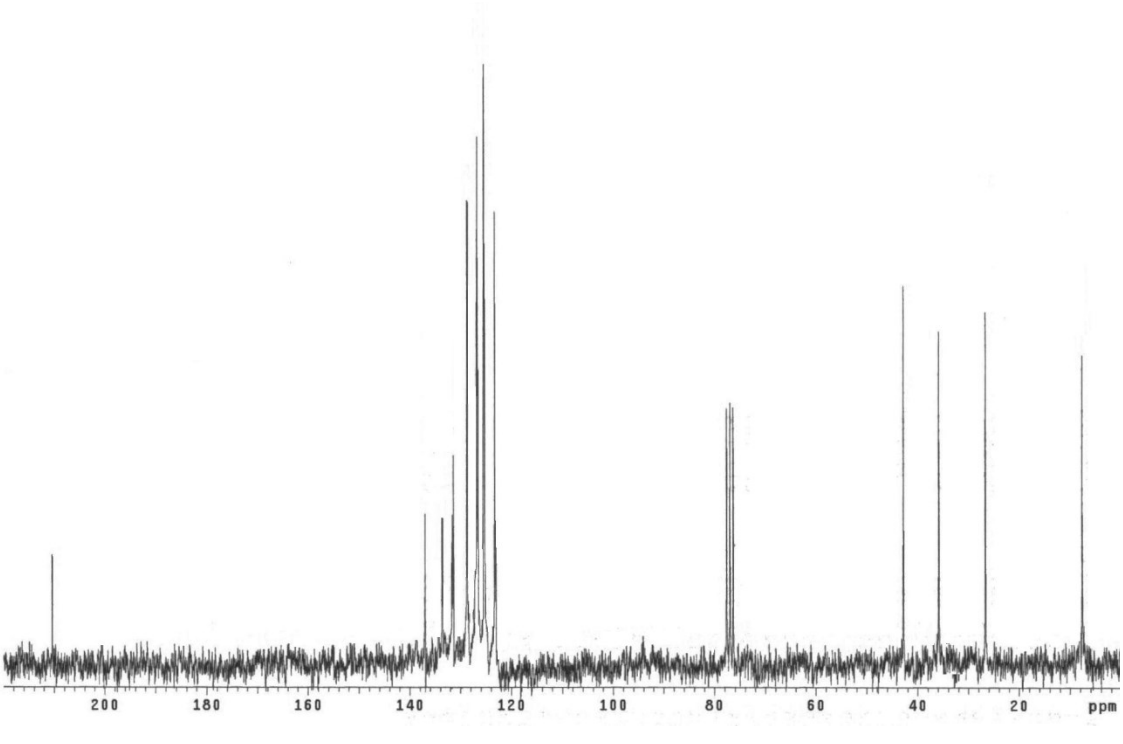


S19. $^{1}$H NMR and $^{13}$C NMR in $CDCl_3$ of **7o**

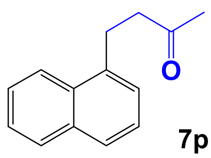

**7p**

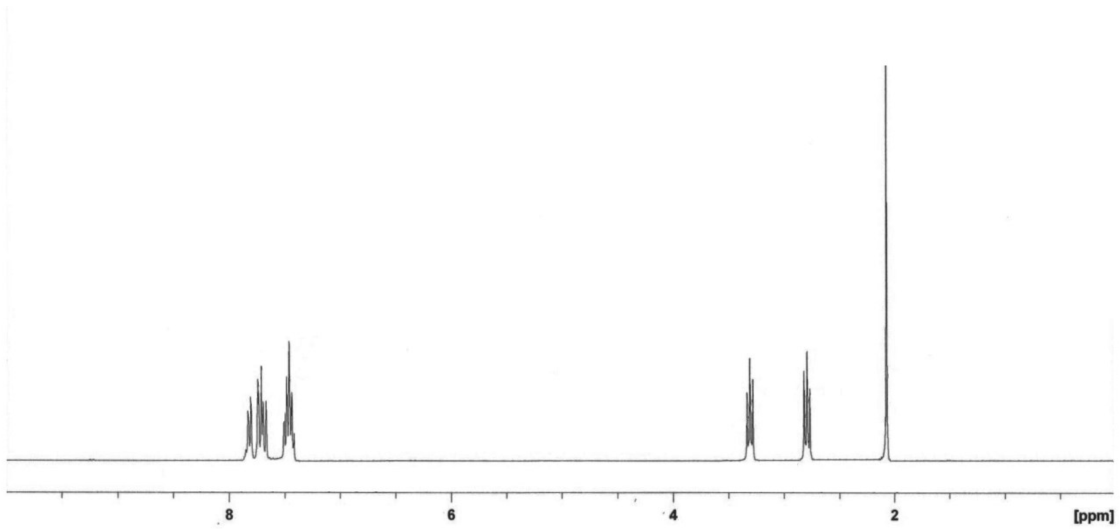


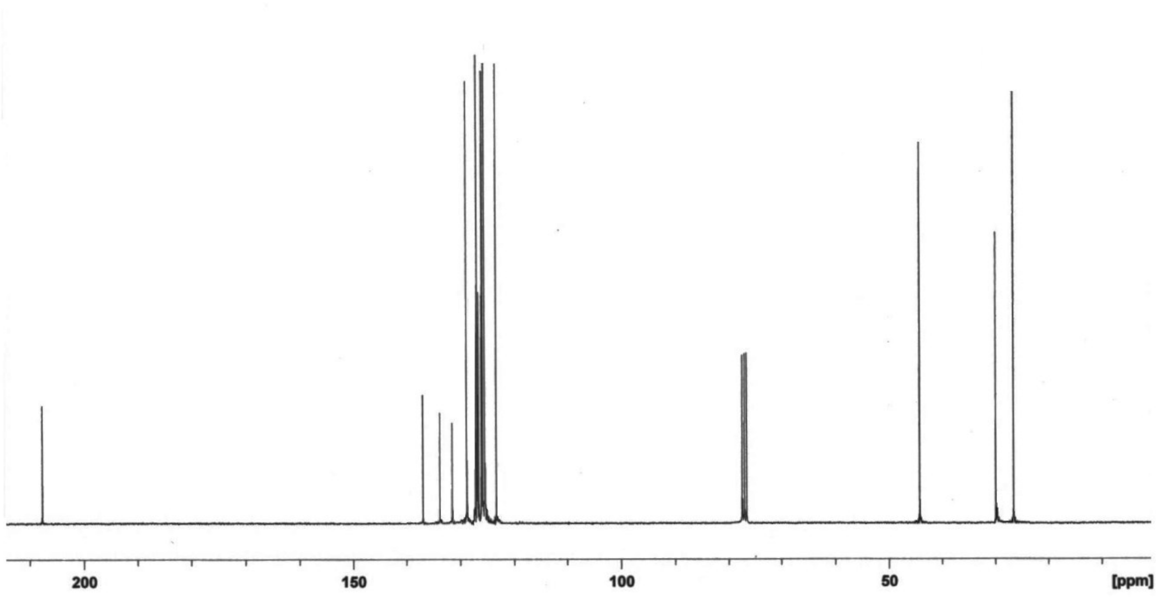


S20. $^{1}H$ NMR and $^{13}C$ NMR in $CDCl_3$ of **7p**

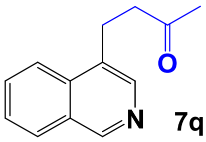

7q

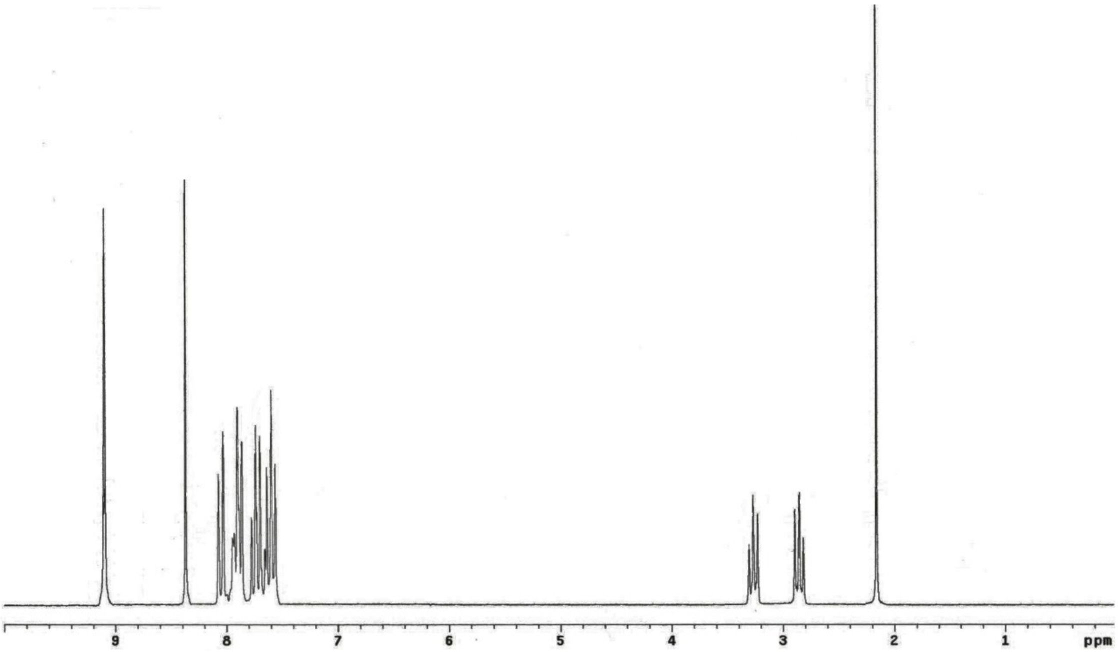


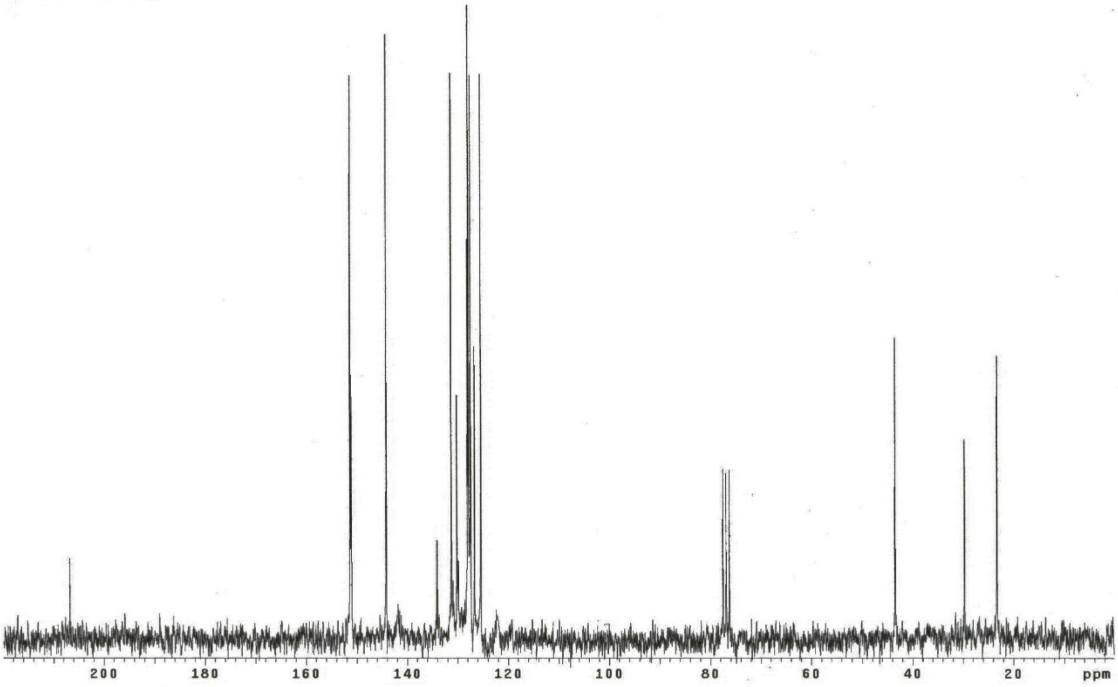


S21. $^{1}H$ NMR and $^{13}C$ NMR in $CDCl_3$ of **7q**

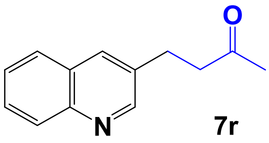

**7r**

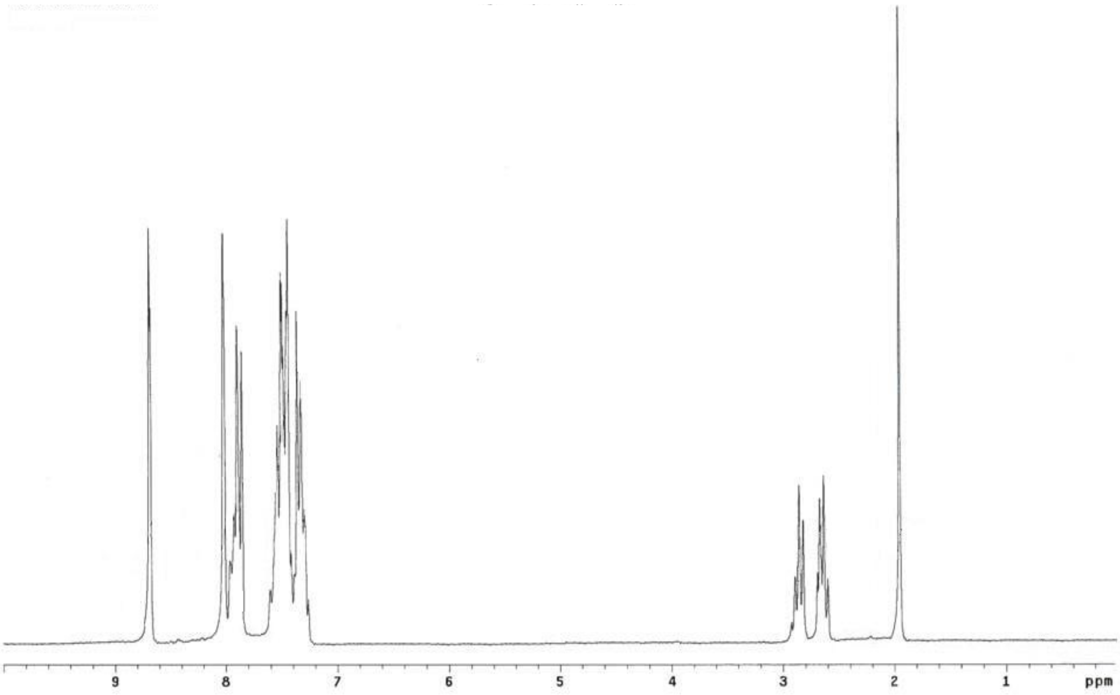


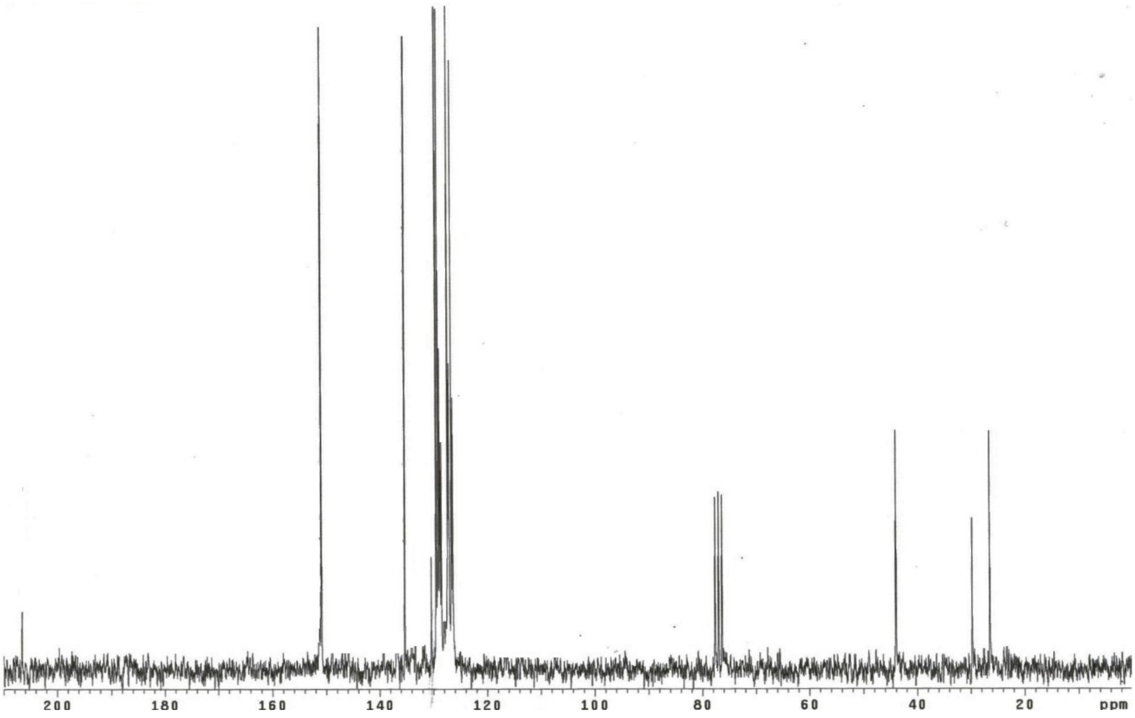


S22. $^{1}$H NMR and $^{13}$C NMR in $CDCl_3$ of **7r**

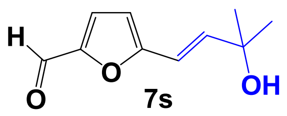


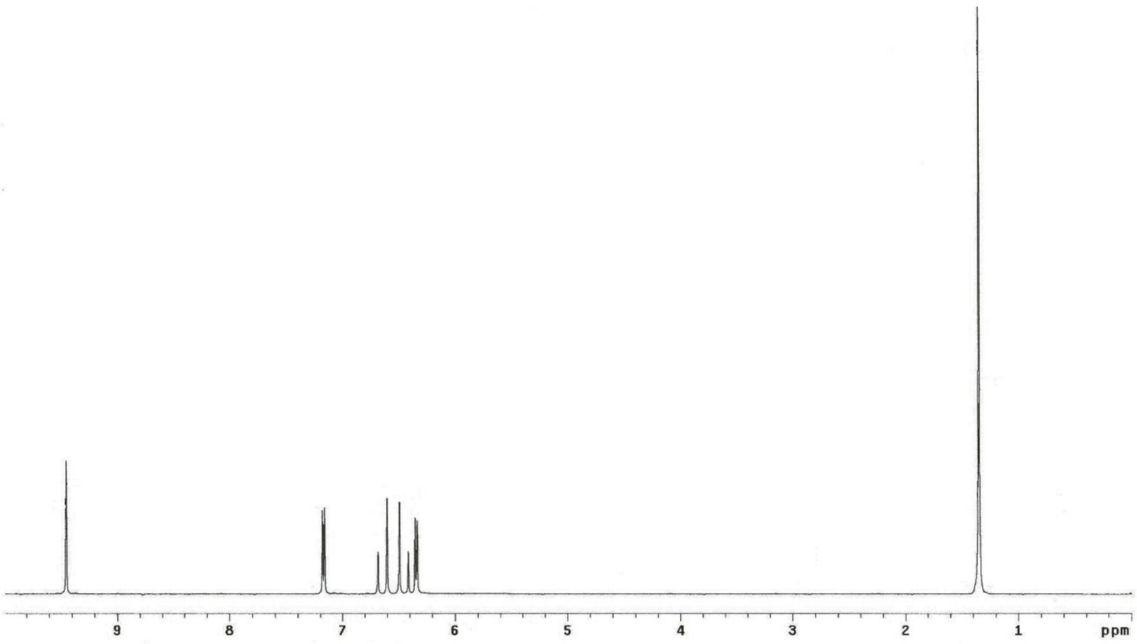


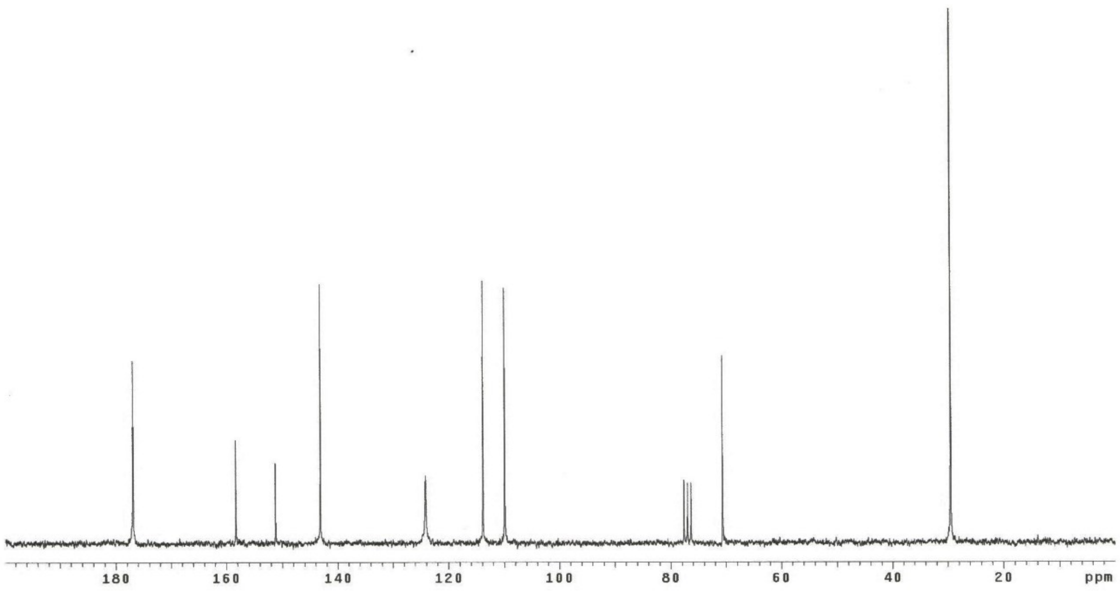


S23. $^{1}H$ NMR and $^{13}C$ NMR in $CDCl_3$ of **76**

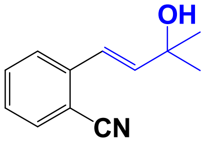


7t

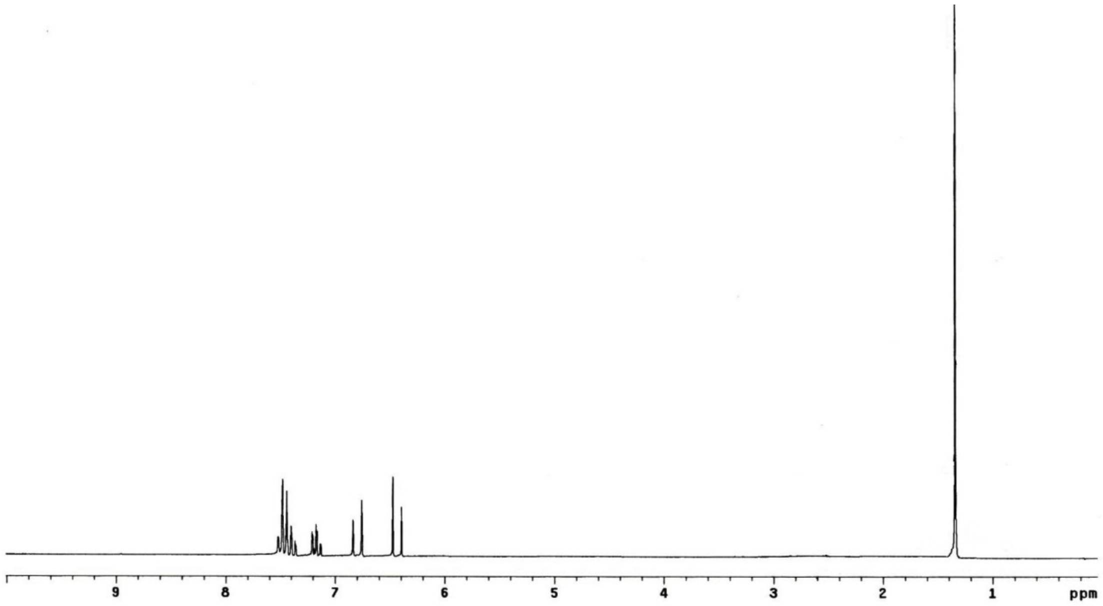


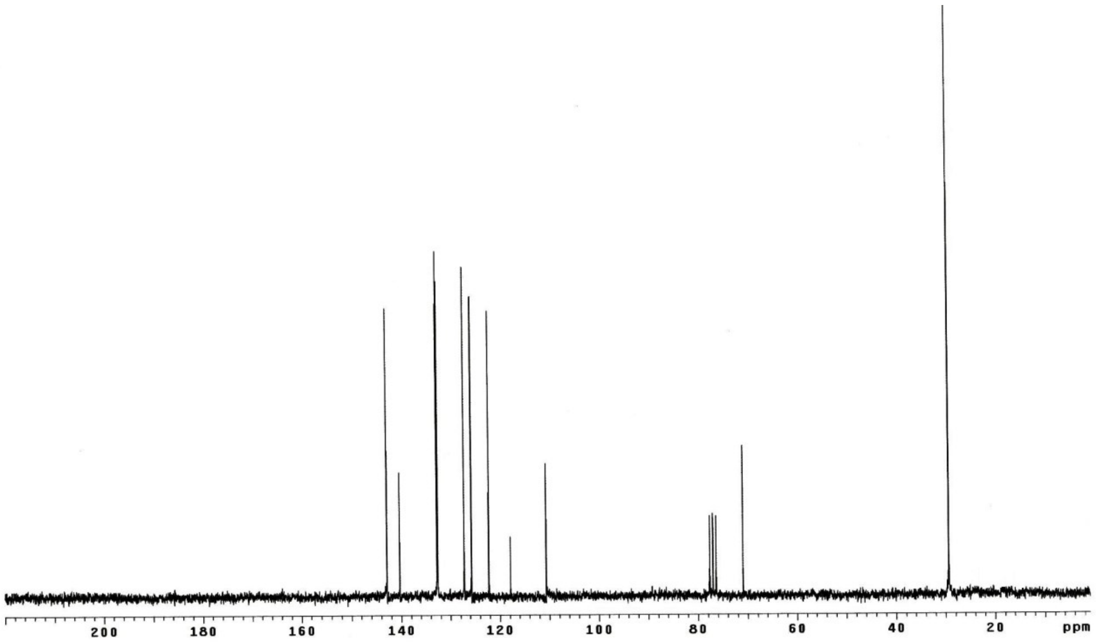


S24. $^{1}H$ NMR and $^{13}C$ NMR in $CDCl_3$ of **7t**

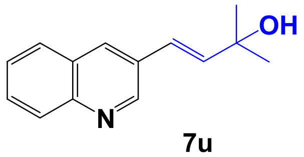


**7u**

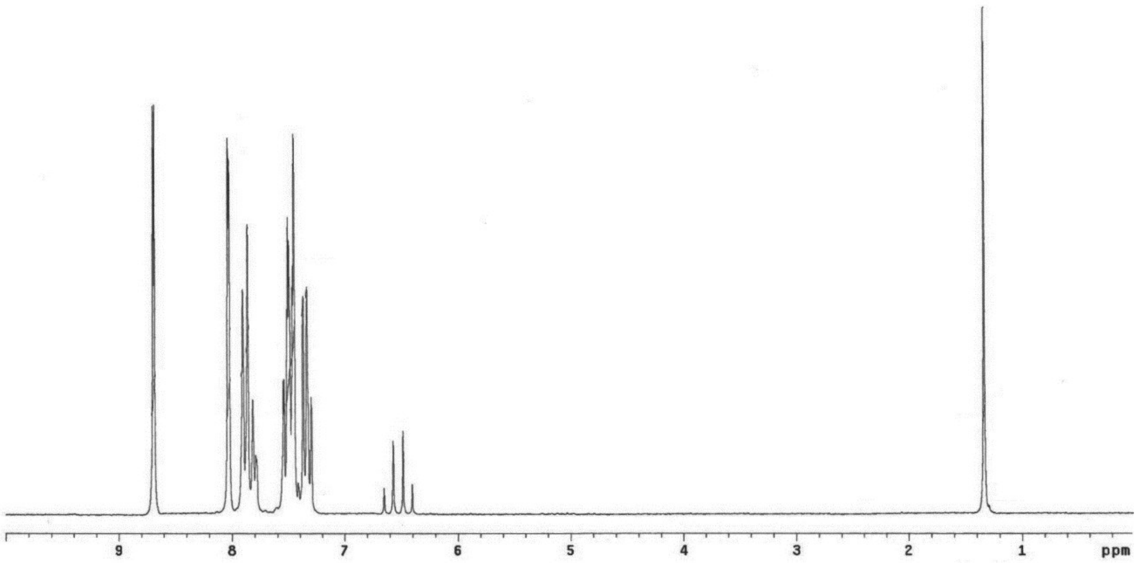


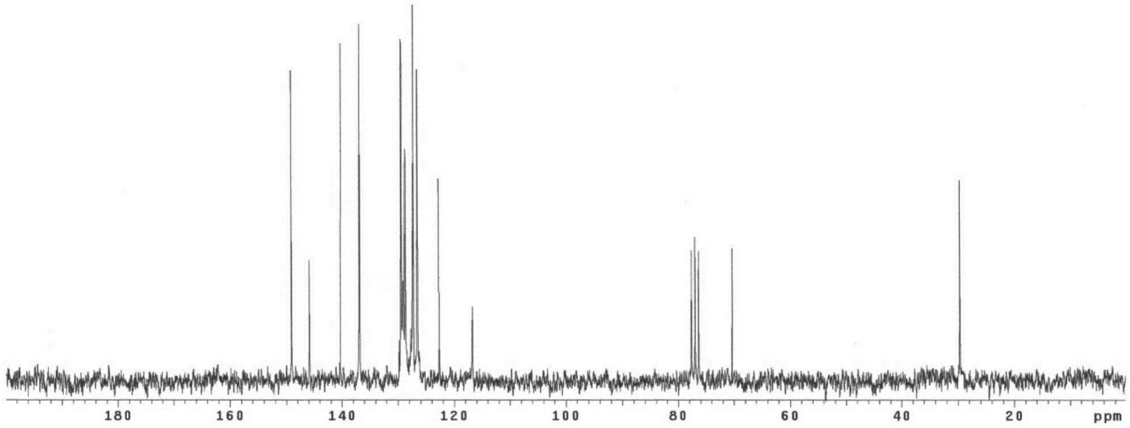


S25. $^{1}H$ NMR and $^{13}C$ NMR in $CDCl_3$ of **7u**

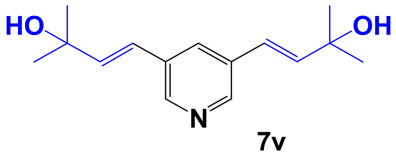


**7v**

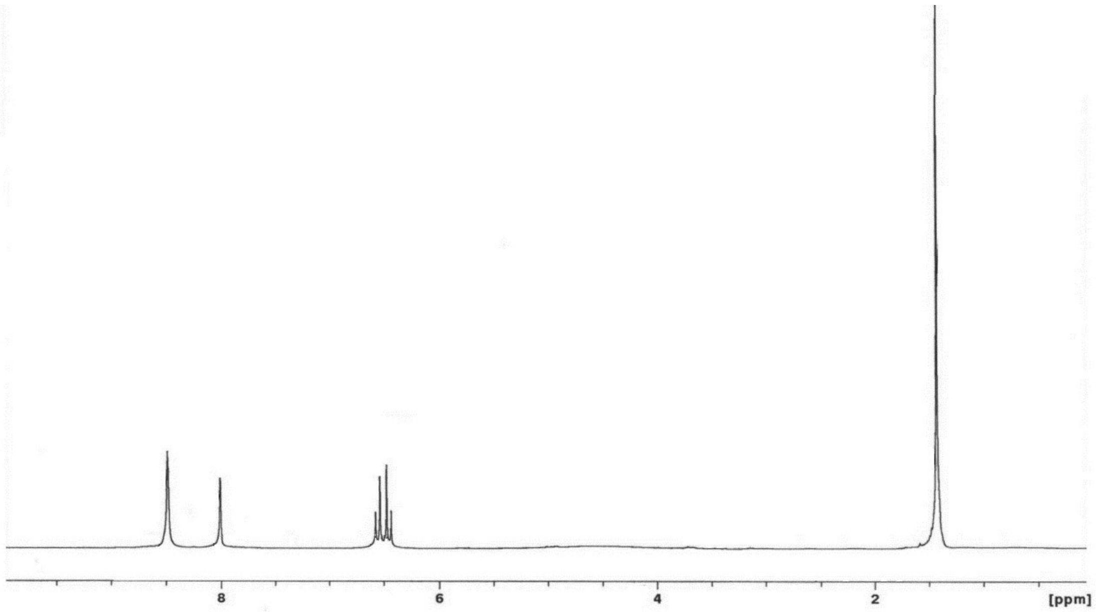


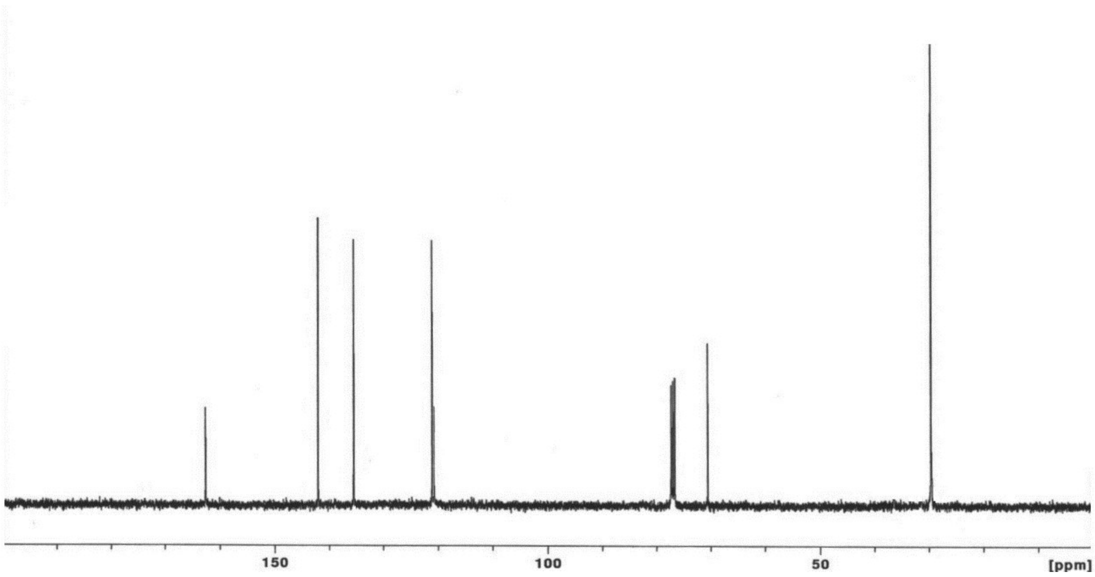


S26. $^{1}$H NMR and $^{13}$C NMR in $CDCl_3$ of **7v**
